# Cross-Species Co-analysis of Prefrontal Cortex Chronic Ethanol Transcriptome Responses in Mice and Monkeys

**DOI:** 10.3389/fnmol.2019.00197

**Published:** 2019-08-13

**Authors:** James W. Bogenpohl, Maren L. Smith, Sean P. Farris, Catherine I. Dumur, Marcelo F. Lopez, Howard C. Becker, Kathleen A. Grant, Michael F. Miles

**Affiliations:** ^1^Department of Molecular Biology and Chemistry, Christopher Newport University, Newport News, VA, United States; ^2^Department of Human and Molecular Genetics, Virginia Commonwealth University, Richmond, VA, United States; ^3^Waggoner Center for Alcohol and Addiction Research, University of Texas at Austin, Austin, TX, United States; ^4^Aurora Diagnostics–Sonic Healthcare, Bernhardt Laboratories, Jacksonville, FL, United States; ^5^Department of Psychiatry and Behavioral Sciences, Medical University of South Carolina, Charleston, SC, United States; ^6^Department of Behavioral Neuroscience, Oregon Health and Science University, Portland, OR, United States; ^7^Division of Neuroscience, Oregon National Primate Research Center, Oregon Health and Science University, Beaverton, OR, United States; ^8^Department of Pharmacology and Toxicology, Virginia Commonwealth University, Richmond, VA, United States; ^9^Department of Neurology, Virginia Commonwealth University, Richmond, VA, United States; ^10^VCU Alcohol Research Center, Virginia Commonwealth University, Richmond, VA, United States

**Keywords:** alcohol, ethanol, genomic, network analysis, WGCNA, microarray, primate, cross-species

## Abstract

Despite recent extensive genomic and genetic studies on behavioral responses to ethanol, relatively few new therapeutic targets for the treatment of alcohol use disorder have been validated. Here, we describe a cross-species genomic approach focused on identifying gene networks associated with chronic ethanol consumption. To identify brain mechanisms underlying a chronic ethanol consumption phenotype highly relevant to human alcohol use disorder, and to elucidate potential future therapeutic targets, we conducted a genomic study in a non-human primate model of chronic open-access ethanol consumption. Microarray analysis of RNA expression in anterior cingulate and subgenual cortices from rhesus macaques was performed across multiple cohorts of animals. Gene networks correlating with ethanol consumption or showing enrichment for ethanol-regulated genes were identified, as were major ethanol-related hub genes within these networks. A subsequent consensus module analysis was used to co-analyze monkey data with expression data from a chronic intermittent ethanol vapor-exposure and consumption model in C57BL/6J mice. Ethanol-related gene networks conserved between primates and rodents were enriched for genes involved in discrete biological functions, including; myelination, synaptic transmission, chromatin modification, Golgi apparatus function, translation, cellular respiration, and RNA processing. The myelin-related network, in particular, showed strong correlations with ethanol consumption behavior and displayed marked network reorganization between control and ethanol-drinking animals. Further bioinformatics analysis revealed that these networks also showed highly significant overlap with other ethanol-regulated gene sets. Altogether, these studies provide robust primate and rodent cross-species validation of gene networks associated with chronic ethanol consumption. Our results also suggest potential novel focal points for future therapeutic interventions in alcohol use disorder.

## Introduction

Alcohol abuse and dependence, clinically defined as “alcohol use disorder (AUD),” represents a serious public health problem in the United States and worldwide. Almost 90,000 Americans die each year from alcohol-related causes, which makes it the third leading preventable cause of death in the United States (Stahre et al., 2014). Approximately 17 million Americans, comprising 7.2% of the national population, had an AUD, according to a 2012 survey (Substance_Abuse_and_Mental_Health_Services_Administration, 2012). Despite the prevalence and severity of the disorder, few effective treatments for alcohol abuse exist today. Advancing our knowledge of the biological underpinnings of alcohol abuse will aid in the development of new, more-effective treatments.

Alcohol use disorder (AUD) is strongly influenced by genetic factors, and recent findings suggest that many genes of small effect size likely contribute to alcohol-related phenotypes (Kerns et al., 2005; Mulligan et al., 2006; Tabakoff et al., 2008; Wolen et al., 2012). Unfortunately, human genome-wide association studies have only identified a handful of genes that are significantly associated with alcoholism, mostly genes involved in alcohol metabolism (Zuo et al., 2014; Hart and Kranzler, 2015). Identifying, validating, and characterizing more of the genes that influence alcohol-related behaviors is vital to our understanding of the molecular mechanisms underlying AUD development and susceptibility.

Ethanol has multiple direct molecular targets, producing alterations in neuronal function and signal transduction (Spanagel, 2009; Ron and Barak, 2016). Moreover, acute and chronic ethanol exposure has prominent actions on gene expression in multiple brain regions, representing a potential underlying mechanism for neuronal plasticity and behavioral sequelae seen with AUD (Kerns et al., 2005; Zhou et al., 2011; Osterndorff-Kahanek et al., 2015; Smith et al., 2016). Such genomic evidence has shown that many genes are expressed as networks with a hierarchical organization (Oldham et al., 2008), whereby the expression of one gene influences the expression of others (Jordan et al., 2004; Snel et al., 2004; Chen et al., 2008). Rather than just identifying lists of single genes, genome-wide expression studies allow application of network-based approaches to functionally organize ethanol responses (Wolen et al., 2012; Smith et al., 2016). Perturbations such as alcohol abuse can alter not only the expression of individual genes but can change the relationship between genes within a network, which could have widespread consequences in animal physiology and behavior (Zhou et al., 2011; Iancu et al., 2013; Smith et al., 2016). Furthermore, network analysis allows the identification of highly connected “hub genes” that may influence the network as a whole and serve as potential targets for therapeutic treatments (Farris et al., 2010; Wolen and Miles, 2012).

To more closely assess brain gene network alterations relevant to the neurobiology of AUD in humans, our studies here have utilized a robust primate model of chronic alcohol consumption in rhesus macaques. Grant et al. (2008) have shown through numerous studies that a schedule-induced polydipsia model can be used to produce reliable high levels of voluntary chronic ethanol consumption, mimicking many aspects of AUD (Baker et al., 2014; Allen et al., 2018). Following a 1-year exposure to ethanol consumption, we identified brain genome-wide expression changes using microarray analysis of anterior cingulate and subgenual cortex. These areas were selected because of their involvement in addiction-related processes, such as reward-based learning, emotion, and motivation (Bush et al., 2000; Allman et al., 2001; Drevets et al., 2008); they have strong connections with other addiction-related brain areas, such as the nucleus accumbens, ventral tegmental area, amygdala, and hippocampus (Freedman et al., 2000; Ongür et al., 2003; Onn and Wang, 2005; Fillinger et al., 2017); and they are dysregulated in addicted states (Goldstein and Volkow, 2002; Volkow et al., 2003; Contreras-Rodríguez et al., 2015). To further validate our results for gene networks related to ethanol consumption, we performed a cross-species analysis of the rhesus data with a chronic exposure model in mice (Becker and Lopez, 2004; Smith et al., 2016; van der Vaart et al., 2017). Such an analysis could provide independent confirmation of networks identified in a single species and prioritize targets for future mechanistic studies.

Our results identify several novel gene expression networks correlating with chronic ethanol consumption in rhesus macaques. Furthermore, we identify several networks conserved across rhesus and mouse chronic ethanol consumption models. These studies identify a novel divergence of networks relating to ethanol consumption vs. being over-represented for ethanol-regulated genes *per se*. Thus, our experiments provide a robust cross-species validation of gene networks relating to ethanol consumption, increasing the probability of translational impact on AUD in humans.

## Materials and Methods

Further details on materials and methods are available in the Supplementary Methods, including about the animal subjects, bioinformatics, cell type enrichment analysis, and collation of homologous genes for the cross-species analysis. No actual animal experiments were conducted during these studies. Tissue samples from rhesus macaques were obtained from the Monkey Alcohol Tissue Research Resource^1^ (MATRR). Mouse microarray data was obtained from experiments to be reported elsewhere (Smith et al.; preprint available at https://www.biorxiv.org, MS ID#: BIORXIV/2019/688267).

### Animals and Ethanol Treatments

All primate procedures were conducted in accordance with the NIH and the Guide for the Care and Use of Laboratory Animals and approved by the Oregon National Primate Research Center IACUC. Tissue from 46 adult male rhesus macaques (*Macaca mulatta*), aged 5 to 11 years, was used in this study. These animals, individually housed at the Oregon National Primate Research Center, were induced to drink ethanol by schedule-induced polydipsia per previously published methods (Grant et al., 2008; Helms et al., 2014), and were then allowed 22 h per day of *ad libitum* access to water and 4% (w/v) ethanol in water for a period of 1 year. Control animals were age-matched within cohorts, were given daily maltose dextran solution (calorically matched to an ethanol drinker) and had access to water during all portions of the experiment. Drinking data was used to calculate a number of drinking phenotypes, which were examined for correlation to gene expression data (see Table S1 for detailed explanation of phenotypes as well as phenotype data). Blood samples were taken approximately every 5 days for measurement of blood ethanol concentrations by gas chromatography (see Grant et al., 2008 for details). Additional blood samples were collected weekly for measurement of the hormones cortisol, adrenocorticotropic hormone (ACTH), testosterone, deoxycorticosterone (DOC), aldosterone, and dehydroepiandrosterone sulfate (DHEA-S) (see Helms et al., 2014 for details of hormone assays). Control animals were treated identically to ethanol consuming animals over the same time period, except that blood draws were less frequent due to lack of blood ethanol determinations. After 1 year of open access to ethanol, necropsy was performed within 4 h of last access to ethanol, and tissue deposited into the MATRR. The animals used for these studies comprised MATRR rhesus cohorts 4, 5, 7a, and 7b. Additional details on primate studies are available in Supplementary Methods and are also detailed on the MATRR website.

Mouse animal studies with chronic intermittent ethanol exposure (CIE) by vapor chamber treatment were conducted at the Medical University of South Carolina (MUSC) and full details of the behavioral and genomic analyses of those studies are being reported separately (Smith et al.; preprint available at https://www.biorxiv.org, MS ID#: BIORXIV/2019/688267). Additional methods are described in Supplementary Methods. Briefly, 47 adult male C57BL/6 mice (Jackson Laboratories; Bar Harbor, ME, USA) were acclimated to the animal facility at MUSC for 2 weeks before undergoing experiments. Mice were aged 7 weeks at commencement of CIE, and 21 weeks at the time of sacrifice. All animals were kept under a 12 h light/dark cycle and given free access to food and water. All studies were conducted in an AALAC-accredited animal facility and approved by the Institutional Animal Care and Use Committee of MUSC. All experimental and animal care procedures met guidelines outlined in the NIH Guide for the Care and Use of Laboratory Animals. For chronic intermittent ethanol exposure (CIE), C57BL/6J male mice were divided into 4 groups: CIE Drinking group (*n* = 12) received inhaled ethanol in the vapor chambers followed by 2-bottle choice drinking (15% ethanol v/v in water; 2 h/d), Air Drinking group (*n* = 11) received only air in the vapor chambers, and 2-bottle choice drinking, CIE Non-Drinking group (*n* = 12) received inhaled ethanol in the vapor chambers but did not drink between CIE cycles, and Air Non-Drinking group (*n* = 12) remained completely ethanol naïve (Becker and Lopez, 2004; van der Vaart et al., 2017).

In this study, we use the two robust models of chronic ethanol exposure described above to examine transcriptome responses in addiction-related brain areas. While some of these responses likely do represent gene expression changes associated with alcohol addiction, our experimental animals cannot necessarily be considered to be addicted to alcohol.

### Sample Preparation and Microarray Analysis

Samples of monkey medial PFC (anterior cingulate and subgenual cortex; Brodmann areas 24, 25, and 32) were obtained from the MATRR. See Supplementary Methods for additional info on sample collection. RNA was extracted from brain tissue using either RNeasy Mini Kit (Qiagen, Valencia, CA; cohorts 4 and 5) or All Prep DNA/RNA/miRNA Universal Kit (Qiagen; cohorts 7a and 7b) following the manufacturer's protocol, and the RNAs from the three brain areas for each animal were pooled to give a broader assay of addiction-related prefrontal limbic areas. RNA samples were tested for degradation using an Experion Automated Electrophoresis System (BIO-RAD, Hercules, CA; cohorts 4 and 5) or a 2100 Bioanalyzer (Agilent Technologies, Palo Alto, CA; cohorts 7a and 7b). The RNA Quality Indices (RQI) of all samples had a mean value of 8.04.

Mice were decapitated on the final drinking day of the fourth CIE cycle, before alcohol was available (following 22 h of abstinence). Brains were immediately removed and dissected as previously described (Melendez et al., 2012) to obtain medial PFC samples. Coronal slices (1 mm) were punch dissected (1.25 mm dia) in medial PFC area and included tissue from prelimbic, infralimbic, and anterior cingulate cortex. Tissues were immediately frozen in liquid nitrogen and stored at −80°C until RNA isolation. Total RNA was extracted using the RNeasy Mini Kit (Qiagen).

Affymetrix GeneChip^®^ Rhesus Macaque Genome Arrays were used to measure monkey gene expression. Monkey RNA samples were processed for microarray analysis in two groups (cohorts 4 and 5, followed by cohorts 7a and 7b) consisting of eight batches processed in a supervised randomization scheme to minimize batch effects, as described previously by our laboratories (Kerns et al., 2005; Wolen and Miles, 2012; Smith et al., 2016). Microarrays for two animals failed quality control standards, and one animal was deemed an outlier by hierarchical clustering of RMA data (see below), leaving 43 animals/arrays (32 ethanol drinking, 11 control).

Affymetrix GeneChip^®^ Mouse Genome 430, type 2 arrays were used to measure mouse gene expression. Sample preparation, hybridization, and array scanning were performed at the MUSC ProteoGenomics Core Facility according to Affymetrix protocols. Batches of samples were processed with treatment groups randomized to minimize batch effects. Array data was transferred to Virginia Commonwealth University in .CEL file format for further analysis.

### Data Normalization

Data analysis was largely performed using The R Project for Statistical Computing^2^, or “R.” Raw microarray expression data from monkeys underwent background correction and quantile normalization in a single group by the Robust Multi-array Average (RMA) method within the affy package for R (Gautier et al., 2004). RMA data was examined for batch effects by principal component analysis. Batch effects were evident for two factors with similar patterns of segregation: microarray processing batch and MATRR cohort. To remove batch effects, RMA data was adjusted using the ComBat method in R (Johnson et al., 2007), with microarray processing batch as the batch factor. Principal component analysis confirmed that ComBat removed the batch effects for both microarray processing batch and MATRR cohort (Figure S1). Rhesus probesets were annotated using data made available by Dr. Robert Norgren^3^ (Spindel et al., 2005).

Affymetrix GeneChip^®^ Mouse Genome 430, type 2 arrays were also analyzed with R. Microarray quality was assessed by RNA degradation, average background, percent present probesets, and multi-dimensional scale plots (first principal component by second principal component). Arrays showing low quality measures, or that were outliers by hierarchical clustering analysis, were removed from the dataset. Background correction and ComBat batch correction were done as with primate arrays.

### Weighted Gene Correlation Network Analysis (WGCNA)

Weighted gene correlation network analysis (WGCNA) was performed on monkey data in this study using the WGCNA package for R (Langfelder and Horvath, 2008). Microarray probesets selected for WGCNA had to pass multiple filters. GeneChip control probesets were excluded. Probesets for which there was no annotation were also excluded from analysis. Very low expression probesets with RMA values <3.5 in ≥80% of samples were excluded. Finally, probesets with a median absolute deviation in RMA value of <0.065 across all samples were excluded from the analysis. This left 31,479 probesets that passed all filters and were used for WGCNA.

Weighted gene correlation network analysis (WGCNA) parameters were left at default except as noted. The soft-thresholding power was set at 6, based on dataset properties described in Langfelder and Horvath (2008). Minimum module size was set to 30. The deep-split value was also chosen based on suggestions in Langfelder and Horvath (2008). In short, WGCNA was run with deep-split values of 0–3, and the results were compared in multi-dimensional scaling (MDS) plots (Figure S2). A deep-split value of 3 was selected, as it provided several modules, most of which were well-segregated on the MDS plot. Hierarchical clustering dendrograms of all probesets and their assignments to modules under deep-split values 0–3 are shown in Figure S3.

Modules created by WGCNA were examined for correlation (Pearson) of the module eigengene (first principal component of expression data) to phenotypic data described in Table S1. For ethanol-related phenotypes and hormone measures, control animals were not considered in the correlations, as no measurements were taken (i.e., control animals were not entered as zero values).

### Ethanol-Responsive Genes

In a separate analysis, monkey microarray RMA expression data was analyzed for ethanol-responsive probesets by the Linear Models for Microarray Analysis (LIMMA) method (Smyth, 2004), using the Multi Experiment Viewer (MeV) software (Saeed et al., 2003). A two-class design was used with treatment group as the factor analyzed and alpha set to 0.01. The probesets used for this analysis were similar to the list of probesets used for WGCNA, except that no median absolute deviation filter was applied (36,243 probesets).

### Candidate Gene Selection

To determine which genes from each ethanol-related module represented the major regulators of ethanol responses, we derived a ranking metric called the ethanol-related hub score (ERHS). This metric was designed to simultaneously capture each probeset's intramodular connectivity and relevance to ethanol consumption or regulation. The ERHS was generated from three values for each probeset: the scaled intramodular connectivity (intramodular connectivity scaled by the gene's maximum possible connectivity in its module; calculated by the WGCNA package), the *p*-value for correlation to the ethanol intake phenotype, and the LIMMA *p*-value for ethanol regulation. The latter two values were subtracted from 1 to preserve directionality, thus:

$$\begin{array}{l} \text{ERHS}=\text{scaled intramodular connectivity}+(1-\text{EtOH intake}\\ \;\text{pvalue})+(1-\text{LIMMA pvalue}) \end{array}$$

Scaled connectivity values allowed ERHS to be compared across large and small modules. All three terms range from 0 to 1, with 1 being the most relevant to our goal. The ERHS values of all probesets in the analysis show a near Gaussian distribution that ranges from 0 to 3 (Figure S4).

### Monkey-Mouse Co-analysis

Expression data from the rhesus macaque chronic ethanol consumption and mouse CIE experiments were combined using procedures described in Supplementary Methods. A list of 10,990 homologous gene pairs was compiled for the co-analysis. A highly significant positive correlation was seen between ranked expression in monkey and mouse for homologous gene pairs (Figure S5).

Expression data from 43 monkeys (32 ethanol drinkers, 11 controls) and 47 mice (23 drinkers, 24 non-drinkers) underwent consensus module analysis (Langfelder and Horvath, 2007) using the WGCNA package for R. To avoid bias in consensus module construction and to ensure the two datasets were on a similar scale, the mouse topological overlap matrix was scaled to the monkey data using the built-in scaling function (Figure S6). The consensus module analysis constructs topological overlap matrices for each species separately (similar to normal WGCNA), and after scaling, these matrices are merged by taking the parallel minimum topological overlap value between the two datasets for each gene-gene comparison. All parameters for module construction were left at default, except for the following, based on the same decision-making process described above for WGCNA. Soft thresholding power was set to 8, deep split value to 3, and minimum module size to 30. Modules underwent hierarchical clustering, and similar modules were merged at a cut height of 0.2, using the merge function in the WGCNA package.

### Data Sharing

Rhesus gene expression data has been uploaded to the Gene Expression Omnibus^4^ (accession number GSE134546) and modules and meta-modules have been uploaded to GeneWeaver^5^ Tables S3–S5, S10 contain all of the data calculated for each rhesus microarray probeset, including annotations, module assignments, RMA values, connectivity data, module membership data, phenotypic correlations, LIMMA results, and ERHS. Table S10 also contains mouse microarray expression data (RMA) for genes matched across mouse and primate arrays.

## Results

### Weighted Gene Correlation Network Analysis

To identify scale-free networks of highly correlated gene expression across rhesus macaque frontal cortex microarray data, we used WGCNA analysis. WGCNA of rhesus macaque expression data produced 30 modules of highly correlated genes (excluding the gray module, which contained uncorrelated probesets not placed into other modules) varying in size from 31 to 3,985 probesets (Table S6). The validity of our module construction method was confirmed by permutation analysis comparing the topological overlap of each module to the mean topological overlap of 100 randomly generated modules of equal size (Table S7). All modules formed by WGCNA had significantly higher topological overlap than the randomly generated modules.

**Figure 1:**
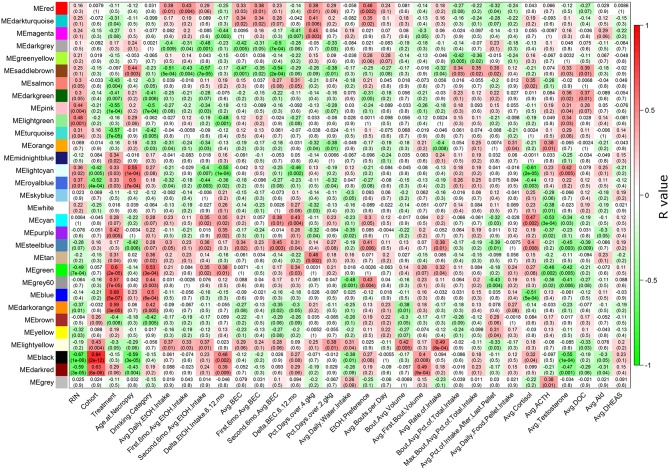
Heatmap showing correlations of each module to each phenotype. The heatmap color scheme is shown at right, with positive correlations being red and negative correlations being green. At each intersection, the top number represents the Pearson correlation *R* value, and the bottom number in parentheses is the *p*-value. Detailed descriptions of phenotypes on the X-axis are found in Table S1.

Multiple WGCNA module eigengenes (Langfelder and Horvath, 2007) showed significant correlations to ethanol-related phenotypes (Figure 1). Seven modules correlated to average daily ethanol intake with a *p* ≤ 0.05: red, darkgray, saddlebrown, orange, royalblue, steelblue, and lightyellow. All RMA data and LIMMA results for each probeset can be found in Table S3, all connectivity and module membership data can be found in Table S4, and all probeset to phenotype correlation data can be found in Table S5. As expected, many phenotypes showed strong correlations among each other. For example, blood ethanol concentrations, ethanol intake, and the percentage of days with high doses of ethanol consumed all had strong positive correlations with one another (Figure S7).

### Ethanol-Responsive Genes and Modules

Linear models for microarray analysis (LIMMA) analysis was used to identify ethanol-responsive genes across ethanol-consuming animals vs. water-only controls. From 36,243 probesets, 2,294 were significantly ethanol-responsive (FDR ≤ 0.01). Hypergeometric overlap analysis showed that six WGCNA modules contained more ethanol-responsive probesets than expected by chance: blue, darkorange, green, grey60, pink, and turquoise (Table S6). Intriguingly, no ethanol-responsive genes were found within the modules correlating with ethanol intake.

### Ethanol-Related Monkey Modules

Six modules are highlighted below, based on their eigengene correlations to ethanol phenotypes (see Table S1 for more detailed descriptions of phenotypes), enrichments with ethanol-responsive probesets, functional enrichments, and relationships to meta-modules discovered in the co-analysis with mouse data. Full ToppGene functional enrichment data for all modules are found in Table S2. The validity of module construction and relevance to ethanol consumption were confirmed through multiple bioinformatics approaches. Modules were independently examined for densely interconnected network structure using external data from multiple sources via GeneMania^6^ analysis. Additionally, modules were examined for a significant positive correlation between their genes' module membership scores and either their correlations to the ethanol intake phenotype or their significance of ethanol-regulation. Finally, modules were examined for overlap with gene sets identified in other publicly available ethanol-related genomic studies (Table S8), using the GeneWeaver web-based resource^7^.

#### Red Module–Myelination

**Figure 2:**
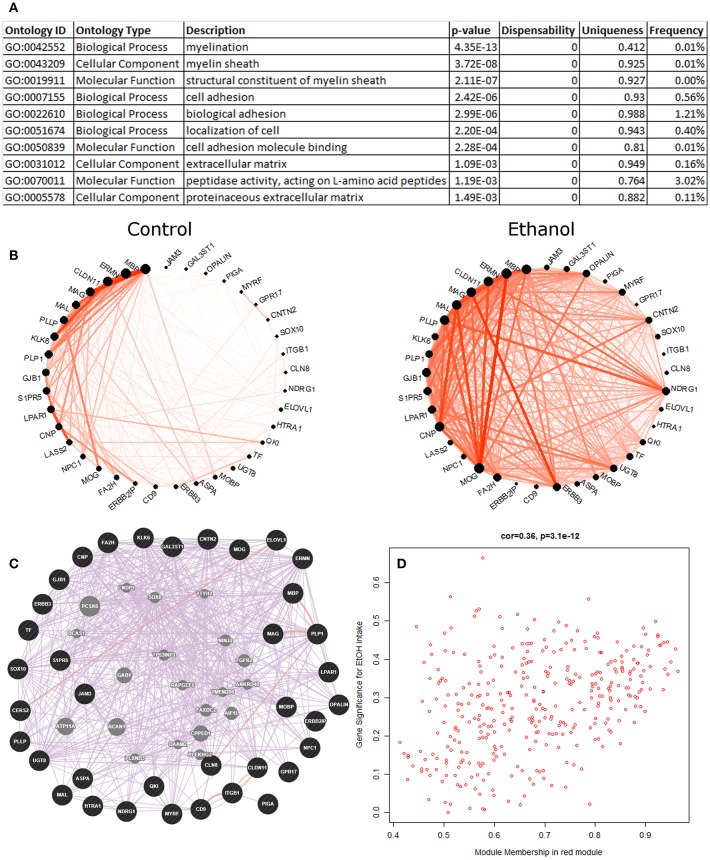
Red module characteristics. **(A)** The top 10 functional enrichments from the REVIGO summary of ToppFun analysis are shown. Almost all pertain to myelination. **(B)** Circle plot showing clear differences in connectivity among the myelin genes within the red module. Note how the gene NDRG1 has little connectivity in the control animals but is a major hub gene in the ethanol animals. **(C)** A network diagram from GeneMania supports the finding that the myelin genes within the red module are highly interconnected, using data from the literature. This network contains several potential hub genes. The arrangement of nodes was modified to better display the number of connections to each query gene (black). Genes added to the network based on connections from the literature are shown in gray. **(D)** Scatterplot of red module membership vs. correlation to the ethanol intake phenotype (gene significance) for each probeset in the red module. Note the highly significant positive correlation.

The eigengene of the red module (353 probesets) had significant positive correlation (Pearson *R* = 0.39, *p* = 0.01) to ethanol intake (Figure 2). The validity of this relationship was confirmed by highly significant relationship between the correlation of red module genes to ethanol intake and their module membership scores (*R* = 0.36, *p* = 3.1E-12; Figure 2D); i.e., genes in the red module most strongly correlated with ethanol intake were those showing the strongest similarity to expression of the module as a whole (module eigengene). The red module eigengene also correlated with BEC, days over 4 g/kg, and ethanol preference (Figure 1). Functional enrichment analysis showed that the most significant enrichment was for genes pertaining to myelination (Figure 2A). Other functions enriched in this module included lipid synthesis, extracellular matrix remodeling, cell migration, and cell morphogenesis: all processes that are peripherally involved in myelination. Massively increased connectivity was observed among myelin genes in the ethanol-drinking animals, as compared to controls (Figure 2B). In particular, *NDRG1* and *ERBB3* showed little connectivity among red module myelin genes in control animals, but these genes became major network hubs in ethanol-drinking animals.

Data from the literature support the construction of the red module, as shown by the highly interconnected network produced by GeneMania (Figure 2C). Additionally, the red module had statistically significant overlap with multiple gene sets from genomic studies of the PFC from acute or chronic ethanol-treated mice (Kerns et al., 2005; Farris and Miles, 2013; Smith et al., 2016; van der Vaart et al., 2017), ethanol-treated mouse liver (Osterndorff-Kahanek et al., 2013), as well as frontal cortex (Lewohl et al., 2000; Liu et al., 2006) and hippocampus (McClintick et al., 2013; Farris et al., 2015) of human alcoholics, as found on GeneWeaver (Table S8).

#### Green Module—Synaptic Transmission

**Figure 3:**
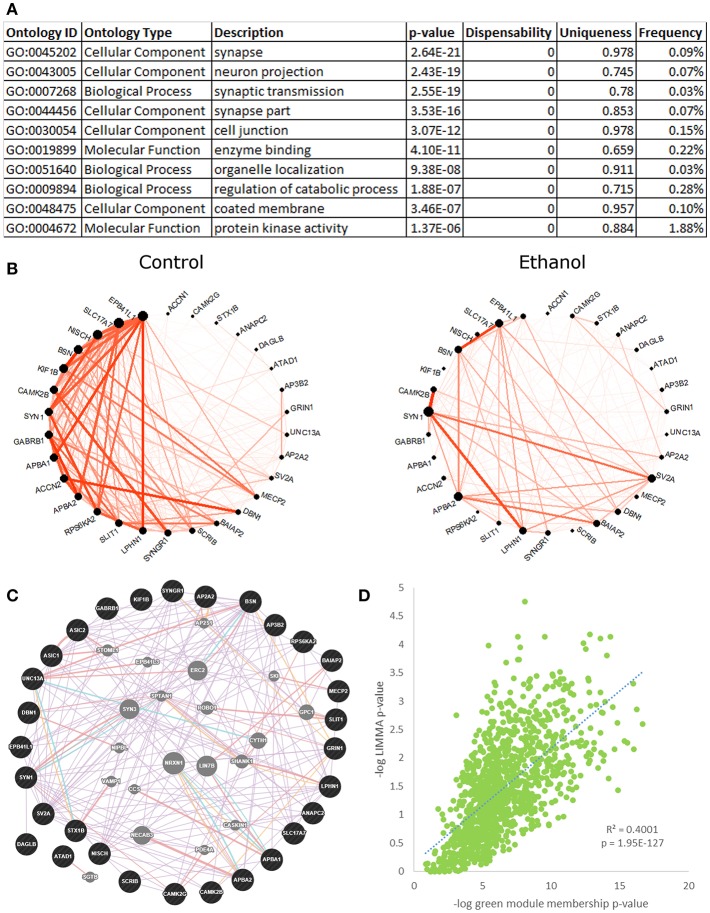
Green module characteristics: synaptic transmission. **(A)** The top 10 functional enrichments from the REVIGO summary of ToppFun analysis are shown. Most pertain to synaptic transmission. **(B)** Circle plot of genes from the “synaptic transmission” ontology hits that were also ethanol-regulated by LIMMA (*p* < 0.01) showed a clear decrease in connectivity in the ethanol-treated animals. **(C)** A network diagram from GeneMania supports the finding that the synaptic transmission genes in **(B)** are highly interconnected, using data from the literature. This network contains several potential hub genes. The arrangement of nodes was modified to better display the number of connections to each query gene (black). Genes added to the network based on connections from the literature are shown in gray. **(D)** Scatterplot of the negative log of green module membership *p*-value vs. negative log of LIMMA *p*-value for each probeset in the green module. Note the highly significant positive correlation.

The green module (Figure 3, Figure S8) was highly enriched for ethanol-responsive genes (hypergeometric test, *p* = 2.53E-82), with 283 out of 1,131 probesets differentially expressed in ethanol-treated vs. control animals, as shown by LIMMA. A highly significant positive correlation between the negative logs of LIMMA *p*-value and module membership *p*-value confirmed the relationship of this module with ethanol; the probesets that were most significantly regulated by ethanol were also the most interconnected probesets within the green module (*R* = 0.63, *p* = 1.95E-127; Figure 3D). The green module eigengene correlated significantly to treatment group, drinking category, ethanol intake during the second 6 months, change in ethanol intake, change in BEC, rate of intake, ACTH levels, and testosterone levels (Figure 1). Functional enrichment analysis of this module showed significant enrichment for genes involved in synaptic transmission and neuronal projection structure and development (Figure 3A). Data from the literature validate this gene network, as shown by the highly interconnected GeneMania network (Figure 3C). A subset of genes from the “synaptic transmission” ontology that were significantly ethanol-responsive showed a substantial reduction in connectivity in ethanol-treated animals, as compared to controls (Figure 3B). *CAMK2B* and *GABRB1* were among the most connected in this subset of genes in control animals, but were only sparsely connected in ethanol-treated animals. A number of receptors and receptor subunits were found in this module, including neurotransmitter receptors such as GABA-A and B receptors (*GABBR1, GABRB1, GABRD*), NMDA receptors (*GRIN1, GRIN2C, GRINA*), histamine receptor (*HRH3*), and neuropeptide Y receptor (*NPY1R*); as well as insulin-like growth factor receptors (*IGF1R, IGF2R*), platelet-derived growth factor receptor (*PDGF*), and neurotrophin receptor (*NTRK3*); in addition to cell adhesion/growth receptors such as ephrin receptors (*EPHB1, EPHB6*) and flamingo-type cadherins (*CELSR2, CELSR3*).

The green module contained several other significant functional enrichments, including chromatin modification (Figure S8A), cell adhesion, intracellular transport, post-translational modification, and GTP catabolism and metabolism. Like the synaptic transmission genes, ethanol-responsive chromatin modification genes showed an overall decrease in connectivity in ethanol-treated animals, as compared to controls (Figure S8B). As a whole, the green module showed significant overlap with gene sets on GeneWeaver from multiple genomic studies of the PFC in acute and chronic ethanol-treated mice (Kerns et al., 2005; Melendez et al., 2012; Wolen et al., 2012; Farris and Miles, 2013; Osterndorff-Kahanek et al., 2013; Smith et al., 2016), as well as from studies of the human alcoholic frontal cortex, hippocampus, and amygdala (Lewohl et al., 2000; Liu et al., 2006; Ponomarev et al., 2012; McClintick et al., 2013; Farris et al., 2015), which lends support to our findings (Table S8).

#### Steelblue Module—Circadian Rhythms

The steelblue module (31 probesets) eigengene correlated significantly with ethanol intake (*R* = 0.3, *p* = 0.05). It also correlated with age, BEC, change in BEC, ethanol preference, rate of intake, intake after the last food pellet, ACTH levels, and testosterone levels. Functional enrichment analysis showed this module was enriched with genes involved in circadian rhythms (Figure S9A). The steelblue module showed decreased connectivity in ethanol-treated animals, as compared to controls (Figure S9B), especially among genes that are components of the circadian molecular clock (*CRY2, PER1, PER2*). These three genes represented major hubs in the steelblue module among control animals and in the GeneMania network (Figure S9C). Significant correlation between module membership and probeset correlation to BEC confirms the association of the steelblue module with ethanol effects (*R* = 0.39, *p* = 0.03; Figure S9D). Functional enrichments for glucocorticoid signaling, synaptic transmission, chromatin modification, ion transport, and vascular processes were also found. GeneWeaver analysis (Table S8) showed significant overlap between the steelblue module and gene sets derived from the PFC of acute and chronic ethanol-treated mice (Kerns et al., 2005; Melendez et al., 2012; Wolen et al., 2012; Farris and Miles, 2013), and from the liver of chronic ethanol-treated mice (Osterndorff-Kahanek et al., 2013).

#### Blue Module—Nuclear Processes

The blue module (Figure S10; 3,172 probesets) was strikingly enriched with ethanol-responsive probesets (1,027; *p* ≈ 0). The blue module eigengene correlated significantly with treatment group, drinking category, and cortisol levels. Functional enrichment analysis revealed a variety of enriched ontologies (likely owing to the large number of genes), including nuclear processes such as DNA repair, chromatin modification, and cell cycle regulation, as well as ubiquitination and calcium regulation (Figure S10A). Functional enrichment analysis on just the 1,027 ethanol-responsive probesets in the blue module revealed strong enrichment for processes involved in RNA processing and transport (Figure S10B). The relevance of this module to ethanol is supported by the highly significant positive correlation between significance of ethanol responsiveness and significance of blue module membership (*R* = 0.73, *p* = 7.62E-135; Figure S10C). The blue module also overlapped significantly (Table S8) with gene sets derived from acute and chronic ethanol-treated mouse PFC (Melendez et al., 2012; Farris and Miles, 2013; Osterndorff-Kahanek et al., 2013; Smith et al., 2016; van der Vaart et al., 2017) and the frontal cortex, hippocampus, and amygdala of human alcoholics (Liu et al., 2006; Ponomarev et al., 2012; McClintick et al., 2013; Farris et al., 2015).

#### Turquoise Module—Protein Synthesis

Like the blue module, the turquoise module (Figure S11) was large (3,985 probesets) and was highly enriched for ethanol-responsive probesets (558; *p* = 3.92E-61). The turquoise module eigengene correlated to treatment group, BEC, days over 4 g/kg, and ethanol preference. Functional enrichment analysis of turquoise module showed highly significant enrichment for genes involved in translation, cellular respiration, and intracellular transport (Figure S11A); all processes that are involved in protein synthesis. Functional enrichment analysis of just the 558 ethanol-responsive probesets showed similar results to the turquoise module as a whole (Figure S11B). Turquoise module genes showed highly significant correlation between significance of ethanol-responsiveness and significance of module membership (*R* = 0.32, *p* ≈ 0; Figure S11C). Additionally, significant overlap was found between the turquoise module and gene sets from genomic studies of acute and chronic ethanol-treated mouse PFC (Kerns et al., 2005; Melendez et al., 2012; Farris and Miles, 2013; Osterndorff-Kahanek et al., 2013; Smith et al., 2016; van der Vaart et al., 2017) and liver (Osterndorff-Kahanek et al., 2013), as well as from the human alcoholic frontal cortex (Lewohl et al., 2000; Liu et al., 2006), hippocampus (McClintick et al., 2013; Farris et al., 2015), and amygdala (Ponomarev et al., 2012).

#### Pink Module—Various Enrichments

The pink module (Figure S12; 291 probesets) was significantly enriched with ethanol-responsive probesets (59; *p* = 1.19E-13). A significant positive correlation was observed between significance of ethanol-responsiveness and significance of pink module membership (*R* = 0.42, *p* = 9.68E-12; Figure S12D). The eigengene correlated significantly with treatment group, drinking category, ethanol intake during the second 6 months, and testosterone levels (Figure 1). Functional enrichment analysis showed that the pink module harbored various enrichments, including Golgi apparatus function, protein synthesis, chromatin modification, and synaptic transmission (Figure S12A). Connectivity analysis of the top 50 genes in the pink module by ERHS showed a reorganization of connectivity with ethanol treatment (Figure S12B). A densely connected network was generated by GeneMania for these 50 genes (Figure S12C). The pink module was also particularly rife with genes having high ERHS; five of the top 25 genes by ERHS from the whole study were in this module (Table 1), including the highest-scoring gene in the study, *SEC23A*, a gene essential for COPII-coated vesicular transport of proteins from the endoplasmic reticulum to the Golgi apparatus. Sec23 regulates the trafficking of GluA1 vs. GluA2 AMPA receptor subunits in rodent striatal medium spiny neurons (Pick et al., 2017), a process important in alcohol and cocaine addiction (Woodward Hopf and Mangieri, 2018). The pink module also overlapped significantly with gene sets derived from acute and chronic ethanol-treated mouse PFC (Wolen et al., 2012; van der Vaart et al., 2017) and the frontal cortex, hippocampus, and amygdala of human alcoholics (Liu et al., 2006; Ponomarev et al., 2012; McClintick et al., 2013; Farris et al., 2015).

**Table 1:** Top 25 genes by ethanol-related hub score (ERHS) from select ethanol-related monkey modules, and from the entire study (far right).

**Red**		**Green**		**Blue**		**Turquoise**		**Pink**		**Steelblue**		**All modules**		
**Gene**	**ERHS**	**Gene**	**ERHS**	**Gene**	**ERHS**	**Gene**	**ERHS**	**Gene**	**ERHS**	**Gene**	**ERHS**	**Gene**	**Module**	**ERHS**
ENPP2	2.56	PHYHIP	2.85	GRIA3	2.82	ARL1	2.73	SEC23A	2.93	LRTM2	2.54	SEC23A	Pink	2.93
EVI2A	2.55	SYN1	2.79	ZNF483	2.78	WTAP	2.71	UBA2	2.91	SLC25A25	2.44	SSR4	Lightyellow	2.92
MOG	2.52	WBP2	2.78	IL28RA	2.77	HMGN4	2.67	ACTR6	2.90	RCC2	2.31	AMY1B	Lightcyan	2.92
ERMN	2.50	LPHN1	2.76	TRIOBP	2.74	ARL1	2.66	ARMCX1	2.85	NAB2	2.30	UBA2	Pink	2.91
MOBP	2.47	FASN	2.74	RBM48	2.74	THAP11	2.65	STRN3	2.78	IER5L	2.26	ACTR6	Pink	2.90
EVI2A	2.45	CDC42BPB	2.73	BMP7	2.73	RBM7	2.63	THAP5	2.71	IER5L	2.25	TNFSF10	Darkturquoise	2.87
MAL	2.45	MAPT	2.69	CTSC	2.72	CDC42	2.60	TANK	2.71	ERF	2.20	GIMAP4	Darkturquoise	2.85
UGT8	2.44	KIAA0427	2.68	PCDH11Y	2.72	MFF	2.60	C5orf44	2.68	CRY2	2.19	ARMCX1	Pink	2.85
TNFAIP6	2.42	MLL	2.68	NUAK2	2.71	EIF5	2.59	ACYP2	2.66	CECR6	2.19	PHYHIP	Green	2.85
GOLGA7	2.39	IQSEC2	2.68	MACC1	2.71	TMED10	2.58	VAMP4	2.66	RTN4R	2.13	MLLT10	Lightcyan	2.83
ENPP2	2.37	HUWE1	2.65	SRSF1	2.71	LYSMD2	2.58	PPARGC1B	2.66	USP2	2.13	GRIA3	blue	2.82
MOBP	2.36	RANGAP1	2.65	SH3BP2	2.68	GLRX3	2.57	MRPL36	2.65	RHOBTB2	2.03	ARGLU1	lightcyan	2.82
LAMP2	2.32	C6orf106	2.64	SFRS11	2.68	PSMD14	2.54	SLC4A10	2.64	ADORA1	1.96	HNRNPC	Brown	2.81
CLDN11	2.32	SEZ6L2	2.64	CYP4V2	2.68	SLC4A10	2.52	NNT	2.63	BRPF1	1.96	RPL13	Lightyellow	2.80
CLDND1	2.31	EPN1	2.61	ZNF713	2.68	NDUFS3	2.52	POMP	2.59	EGR1	1.94	SYN1	Green	2.79
ERMN	2.28	SV2A	2.61	SFRS11	2.67	MRPL47	2.51	RCHY1	2.58	PER1	1.75	MYL9	Darkgrey	2.79
MOG	2.28	AGPAT1	2.60	RINL	2.65	WTAP	2.50	PNPLA8	2.58	DGAT2	1.61	STRN3	Pink	2.78
TNFAIP6	2.27	POLR2A	2.60	TCTA	2.64	SMIM8	2.49	TCEA1	2.57	EGR1	1.61	ZNF483	Blue	2.78
CNP	2.25	CD99L2	2.58	MXRA7	2.64	UQCRC2	2.49	CACYBP	2.55	SETD1B	1.54	WBP2	Green	2.78
ENPP2	2.23	C9orf86	2.57	XIAP	2.64	TOMM22	2.49	TMED7	2.55	ADRB1	1.46	IL28RA	Blue	2.77
PPP1R14A	2.23	MICAL3	2.57	PGAP1	2.63	PSMC6	2.48	SLMO2	2.55	DBP	1.44	LPHN1	Green	2.76
C21orf91	2.22	ATP1A3	2.56	FOXK1	2.63	PSMB6	2.48	DPY30	2.55	CNNM2	1.37	ZFR	Brown	2.75
PLLP	2.21	SRRM3	2.55	SUGT1	2.62	RNF138	2.48	EFHA2	2.54	MXRA5	1.32	FASN	Green	2.74
GPR37	2.21	SLC17A7	2.55	HBS1L	2.62	DRG1	2.46	ARL6IP5	2.52	SERPINE2	1.31	TRIOBP	Blue	2.74
CLMN	2.21	GDI1	2.54	OR7D2	2.61	UBE2N	2.46	SRP9L1	2.51	FAM160A2	1.30	RBM48	Blue	2.74

*Retracted hypothetical genes and pseudogenes have been removed from the table. Repeated gene symbols indicate separate probesets for the same gene*.

### Cell Type Enrichment Analysis

Cell type enrichment analysis was performed to find modules that were enriched for genes with selective expression in various brain cell types. Fourteen out of thirty modules showed such an enrichment (Table S9). Eight modules were enriched for neuronal markers (purple, green, salmon, cyan, yellow, white, brown, and darkred), four were enriched for glial markers (red, tan, magenta, and greenyellow), and two were enriched for vascular markers (darkgray and darkturquoise). Among these modules, red (enriched for oligodendrocytic markers) and darkgray (enriched for vascular mural cell markers) correlated with ethanol intake, and the green module (enriched for neuronal markers) was enriched with ethanol-responsive genes, suggesting that chronic ethanol exposure alters gene expression in these cell types. Previous studies have shown that coexpression networks from brain samples are often enriched for markers of a particular cell type (Oldham et al., 2008; Miller et al., 2010). Thus, these findings validate our module construction method. Additionally, the cell type enrichment results largely agree with the functional enrichment results from ToppGene, validating our compiled cell type enrichment dataset.

### Monkey-Mouse Co-analysis

In order to enhance the translational potential of our study, a cross-species co-analysis was performed using rhesus expression data and microarray data collected from studies on C57BL/6J mice exposed to chronic ethanol vapor exposure and consumption (Smith et al.; preprint available at https://www.biorxiv.org, MS ID#: BIORXIV/2019/688267). Such an analysis could identify ethanol-related gene networks conserved across model systems, with increased relevance to AUD. Modules discovered in this co-analysis have been termed “meta-modules” to avoid confusion with monkey-only modules. Consensus module analysis found 15 meta-modules of genes with highly correlated expression patterns across both species. A spreadsheet with meta-module assignments, RMA values, and meta-module membership values is found in Table S10. These meta-modules were examined for correlation to ethanol-related phenotypes in both monkeys and mice (Figures S13, S14). Complete functional enrichment results for meta-modules are found in Table S11. Cross-tabulation of monkey modules with meta-modules showed that 14 out of 30 monkey modules had a correlate meta-module, as shown by significant overlap in a hypergeometric test (Figure S15), indicating their validity in more than one model system. Every meta-module showed significant overlap with at least one monkey module discovered in this study (Figure S15, Table S12). Five of the ethanol-related monkey modules had correlates in the co-analysis: blue, green, pink, red, and turquoise. Details of the five meta-modules most strongly overlapping with these monkey modules are described below.

The most significant overlap was between the meta-black module (114 genes) and the red monkey module, with 88 genes in common (*p* = 1.91E-152, hypergeometric test). Like the red module, the meta-black module contained significant enrichment for genes involved in myelination (Table S11). While the meta-black module eigengene did not correlate with ethanol intake in monkeys (as did the red monkey module), it did correlate with treatment group, drinking category, average BEC, average bout volume, and testosterone levels (Figure S13). The meta-black module eigengene positively correlated with baseline drinking in mice but not to drinking after chronic ethanol vapor exposure (Figure S14).

The meta-green module (124 genes) shared 60 genes with the green monkey module (*p* = 7.6E-46). Meta-green was enriched for genes involved in cell projection assembly, ion channel function, synapses, immune function, and cytoskeleton assembly (Table S11), processes that are important for neuronal development and synaptic transmission. The meta-green module eigengene correlated with treatment group, drinking category, and testosterone levels in monkeys (Figure S13), as well as with drinking at baseline and after CIE in mice (Figure S14).

The meta-turquoise module (594 genes) bore resemblance to the turquoise monkey module, with 280 genes in common (*p* = 7.18E-111), as well as to the blue monkey module, with 109 genes in common (*p* = 9.8E-16). Meta-turquoise was most enriched for genes involved in RNA processing, ubiquitination, ribosomes, and transcription factor activity (Table S11). This meta-module's eigengene correlated to the change in ethanol intake, change in BEC, and days of heavy drinking in monkeys (Figure S13), as well as to drinking after the fourth cycle of CIE in mice (Figure S14).

The meta-yellow module and the blue monkey module shared 80 genes (*p* = 3.23E-49). Meta-yellow harbored enrichment for genes involved in RNA splicing, helicase activity, translation regulator activity, and protein kinase activity (Table S11). The meta-yellow eigengene correlated significantly with treatment group, drinking category, change in ethanol intake, and testosterone levels in monkeys (Figure S13), as well as with treatment group, baseline drinking, and drinking after CIE in mice (Figure S14).

Forty-four genes were shared between the meta-blue module (216 genes) and the pink monkey module (*p* = 1.05E-43). Functional enrichment analysis showed that meta-blue was enriched for genes involved in vesicular transport, Golgi function, post-translational modification, and synapse components (Table S11). The meta-blue module eigengene correlated significantly with baseline and post-CIE drinking in mice (Figure S14), as well as with drinking category and the change in drinking from the first to the second 6 months in monkeys (Figure S13).

Finally, the meta-pink module shared 29 genes with the purple monkey module (*p* = 2.01E-35). Although that monkey module did not show over-representation for ethanol-regulated genes or significant eigengene correlation with 12-month ethanol consumption, the purple module did show a significant positive correlation with the change in drinking from the first 6 months vs. the second 6 months of consumption. Further, the meta-pink module showed a significant correlation with the change in ethanol consumption for the last cycle of CIE (CIE4% change baseline). This suggests that this meta-module/monkey module pair may be related to the escalation of ethanol consumption. This is of interest given the significant functional over-representation of the pink meta-module for genes relating to synaptic transmission, nervous system development and neuronal projection morphogenesis (Table S11).

## Discussion

In this study, we have performed network analysis of genome-wide expression data from an addiction-related brain area of chronically ethanol-drinking primates. Furthermore, we have co-analyzed this monkey data with similar data from another robust animal model of chronic ethanol exposure and consumption, CIE-treated mice. We have identified several functional categories of genes whose response to ethanol is conserved across species and ethanol treatment paradigms, including myelination, neuronal development, synaptic transmission, Golgi apparatus function, and several aspects of gene expression, to name a few. Additionally, we have created a novel multifactorial method for ranking genes that our data suggest to have the best potential for further mechanistic investigation and possible identification of therapeutic targets for AUD (Table 1). Our findings have been validated by several methods, both intrinsic and extrinsic to our own analysis. Altogether, these studies provide significant new information about prefrontal cortex adaptations associated with ethanol consumption.

### Correlation to Ethanol Intake vs. Enrichment With Ethanol-Responsive Probesets

We used two different criteria to characterize WGCNA module relationships to ethanol: correlation of the module eigengene to ethanol intake, and enrichment of the module with ethanol-responsive probesets. Intriguingly, we found very little overlap between these two criteria. This may be due to differences in the data that are considered when calculating the two parameters. For the calculation of correlation to ethanol intake, data from control animals was not available (control animals were not entered as zero values), reducing the number of animals contained in the phenotypic correlations to 32. Thus, module eigengene correlations to drinking phenotypes identified genes that vary expression with *how much ethanol-drinking animals consumed*. On the other hand, the LIMMA analysis examined differences in gene expression between the control and ethanol-drinking groups. As such, these ethanol-responsive modules highlight genes that vary expression with *whether the animal had the opportunity to drink ethanol*.

Alternatively, modules with expression correlated to ethanol intake could represent gene networks that *regulate drinking behavior*, whereas modules that are enriched with ethanol-responsive genes are *regulated by drinking*. This interpretation is supported by findings from the cross-species co-analysis. Similar to segregation of monkey modules correlated to ethanol intake vs. enriched with ethanol-regulated genes, it was evident that most meta-modules correlated with either baseline drinking or drinking after CIE in mice, not both (Table S13). For example, the meta-black module was enriched for myelin genes, and its eigengene correlated to baseline drinking in mice. This observation is in agreement with previous findings that, across the BXD recombinant inbred panel of mice, basal myelin gene expression correlated to initial sensitivity to ethanol (Kerns et al., 2005; Farris and Miles, 2013), which would influence the propensity to drink. In a similar manner, the red monkey module was also enriched for myelin genes, and its eigengene correlated to ethanol intake, suggesting an influence of this module on the propensity of monkeys to drink ethanol, if the role of myelin gene expression in initial sensitivity to ethanol is conserved between mice and monkeys.

There was also a dichotomous relationship between the measure of ethanol-relatedness and the type of functional enrichments observed. Monkey modules that correlated to ethanol intake behavior were often functionally enriched for ontologies of *emergent* cellular processes like myelination, synaptic transmission, circadian rhythms, steroid signaling, and vascular processes, suggesting that expression of these genes is dependent upon the amount of ethanol consumed, or that they influence the organism's propensity to drink ethanol. This is in contrast to modules enriched with ethanol-responsive genes, which contained functional enrichments related to *core* cellular processes, such as translation, cellular respiration, intracellular transport, cell cycle, chromatin modification, Golgi function, and RNA processing, which suggests these processes could be regulated by the presence or absence of ethanol.

Finally, it is possible that withdrawal from ethanol had some influence upon our detection of ethanol-regulated genes and thus might skew the relationship with gene expression correlating with consumption. All monkeys underwent necropsy within 4 h of their last access to ethanol and thus could have been undergoing mild withdrawal.

### Biological Functions Related to Chronic Ethanol Drinking

Perhaps the most clear-cut and consistent finding across our various analyses is the association of a network of myelin genes with ethanol intake. Elucidating ethanol's effects on myelin has been an active area of research for some time. In fact, a recent MRI study showed white matter reductions in the same four cohorts of monkeys used in this study (Kroenke et al., 2014). Postmortem studies of human alcoholic brains have shown sizeable reductions in white matter (de la Monte, 1988; Harper, 2009), diffusion tensor imaging has been used to show microstructural disruptions in myelin integrity *in vivo* in human alcoholics (Pfefferbaum et al., 2000), and these white matter defects have been associated with neurological deficits in alcoholics (Sullivan and Pfefferbaum, 2005; Colrain et al., 2011). Furthermore, studies in mice show correlations between basal myelin gene expression and behavioral sensitivity to ethanol (Kerns et al., 2005; Farris and Miles, 2013) and adolescent binge exposure decreased myelin gene expression in adolescent mice (Wolstenholme et al., 2017). Additionally, adolescent binge drinking or adult alcohol dependence induction in rats caused a reduction in the white matter of the anterior corpus callosum, as well as degradation of myelin basic protein in the PFC (Vargas et al., 2014). Pertinently, previous genomic studies of ethanol have found functional enrichment for myelin among their lists of ethanol-regulated genes (Lewohl et al., 2000; Kerns et al., 2005; Liu et al., 2006; McClintick et al., 2013). It has been suggested that in addition to likely toxic effects of ethanol on myelin expression, underlying differences (e.g., genetic) in basal myelin expression might be a risk factor for ethanol consumption, possibly by altering acute sensitivity to ethanol (Farris and Miles, 2013).

In agreement with these previous findings, we found that chronic ethanol has profound effects on a network of myelin genes in primates and rodents. The red monkey module was enriched for genes involved in myelination, and its eigengene correlated significantly to ethanol intake. Several findings support and confirm the construction of this module and its association with ethanol. The red module had significant overlap with multiple alcohol-related gene sets discovered in studies by other researchers using different species and alcohol treatment paradigms. Also, co-analysis of monkey and mouse expression data led to construction of a cross-species meta-module that was also enriched for myelin genes and correlated to ethanol-related phenotypes in both species. Several of the top 25 genes by ERHS in the red module were canonical myelin genes, including *ENPP2, MOG, ERMN, MOBP, MAL, UGT8, CLDN11, CNP*, and *PLLP* (Table 1). Perhaps somewhat unexpectedly, myelin gene expression was not significantly up- or down-regulated in our rhesus data. However, chronic ethanol drinking was associated with profoundly increased connectivity among myelin genes in the red module (Figure 2B), suggesting ethanol might have altered a common regulator of these genes. In addition, ethanol induced the emergence of new hub genes in the red module (see NDRG1, ERBB3, and CNTN2). It is possible that these changes in myelin network connectivity are a compensatory response to a toxic effect of ethanol on myelin, as has been suggested by some prior studies.

These ethanol-induced hub genes in the red module could represent targets for pharmacological treatments for alcohol abuse, given their ethanol-responsive major change in connectivity with myelin genes. The gene *NDRG1* had an ERHS of 1.71, and showed a striking difference in connectivity between ethanol and control animals. *NDRG1* encodes a cytoplasmic signaling protein that plays a role in development and maintenance of myelin (King et al., 2011), and mutations in this gene cause demyelinating disorders (Kalaydjieva et al., 2000; Hunter et al., 2003). Our previous studies have shown that acute and chronic ethanol treatment increase *NDRG1* expression in mice (Kerns et al., 2005; Farris and Miles, 2013; Smith et al., 2016). Acute ethanol treatment also increases phosphorylation of NDRG1 (Costin et al., 2013). Similar to *NDRG1, ERBB3* was a major hub among myelin genes in the ethanol-treated monkeys and had an ERHS of 1.98 in the red module. This gene encodes an epidermal growth factor receptor important in the development of myelin. While *ERBB3* has not yet been directly implicated in ethanol behaviors, it interacts with ethanol in the development of tumors *in vitro* (Luo and Miller, 2000).

Another striking finding from our analyses is that a large network of genes involved in neurodevelopment and synaptic transmission was regulated by chronic ethanol, and many chromatin modification genes were organized into the same module. It has been known for some time that ethanol alters neuronal function, interacting with and altering properties of several neurotransmitter receptors and ion channels (Spanagel, 2009). The green module was heavily enriched for neuron-specific functional categories, particularly those involved in neuronal process development, such as “neuron projection” (120 genes, *p* = 2.43E-19), and “dendrite” (70 genes, *p* = 4.03E-15), as well as synaptic development and function, such as “synapse” (98 genes, *p* = 2.64E-21) and “synaptic transmission” (108 genes, *p* = 2.55E-19). Among the top 25 green module genes by ERHS were several involved in neuronal process development/structure and cytoskeletal/extracellular matrix reorganization, including *SYN1, LPHN1, CDC42BPB, MAPT, IQSEC2, C6orf106, SEZ6L2, CD99L2*, and *ATP1A3* (Table 1). *SYN1* (synapsin I) encodes a vesicular phosphoprotein involved in synaptogenesis and neurotransmitter release that is a known target of Protein Kinase A (PKA) and is phosphorylated in response to ethanol treatment (Conti et al., 2009). *PRKAR1B*, a regulatory subunit of PKA, is also found within the green module with high ERHS (2.21), along with two A kinase anchor proteins: *AKAP1* and *AKAP8L* (ERHS 1.99 and 2.03, respectively). An extensive body of evidence links PKA activity to ethanol behaviors (see Ron and Barak, 2016 for review). Also within the top 25 genes by ERHS in the green module were three genes involved in lipid synthesis/metabolism (another process important for developing neurons): *PHYHIP, FASN*, and *AGPAT1*. Several more of the top genes in the green module by ERHS were involved in vesicular processes, which are important for neuronal function and synaptic transmission, including *EPN1, SV2A, MICAL3, SLC17A7*, and *GDI1*.

The green module also harbored significant enrichment for chromatin modification genes (62 probesets, *p* = 1.97E-8). *WBP2, MLL, POLR2A, SRRM3*, and *BRD3* all had ERHS above 2.5 (Table 1). The organization of these genes in an expression module with synaptic transmission genes suggests a possible reciprocal relationship between ethanol regulation of synaptic transmission and chromatin modification. This is consistent with a growing body of literature suggesting epigenetic regulation of gene expression by ethanol in brain (Wolstenholme et al., 2011, 2017; Kyzar et al., 2017).

RNA processing and regulation of transcription were functions enriched within the highly ethanol-responsive blue module. Ethanol-induced alterations of gene transcription have been observed previously (Saba et al., 2006; Ponomarev et al., 2012). Three genes involved in RNA splicing/processing were among the top 25 blue module genes by ERHS (*RBM48, SRSF1*, and *SFRS11*), and five were involved in regulation of transcription (*ZNF483, MACC1, SH3BP2, ZNF713*, and *FOXK1*). A recent RNA-seq expression network study in area 32 and central nucleus of the amygdala (CeA) of chronically drinking monkeys, from the same cohorts used in the analysis here, found functional enrichment for genes involved in RNA splicing in CeA (and other forms of transcriptional regulation) as seen with our analysis in PFC (Iancu et al., 2017). However, there was surprisingly no significant degree of overlap between the gene set from area 32 correlating with ethanol consumption in that study (see Supplementary Table 7 in Iancu et al., 2017) with our current analysis (Table S3). Furthermore, Iancu et al. found minimal significant functional over-representation in the genes from area 32 correlating with consumption. Methodological differences likely explain these differences, with our analysis pooling RNA from three anatomically similar areas of medial PFC and using microarrays, compared to focus on area 32 and use of RNAseq by Iancu et al.

Also having high ERHS in the blue module were four genes involved in neuronal development/function: *GRIA3, TRIOBP, BMP7*, and *PCDH11Y*. *GRIA3*, which had the highest ERHS in the blue module (2.82), encodes the glutamate ionotropic receptor AMPA type subunit 3. Growing evidence supports a role for the AMPA receptor in the rewarding properties of alcohol (Cannady et al., 2016; Salling et al., 2016), and expression levels of *GRIA3* splice variants (which differ in several ion channel properties) correlate to ethanol intake and BEC in chronic alcohol-drinking monkeys (Acosta et al., 2011). *BMP7* interacts with ethanol in cell culture experiments. Hepatocytes cultured on a printed array of BMP7 protein are protected from ethanol-induced apoptosis (Wilkemeyer et al., 1999), and ethanol inhibits the morphological changes and cell adhesion induced by BMP7 exposure in cultured neuroblastoma/glioblastoma cells (Jones et al., 2010).

Many genes involved in protein translation and cellular respiration were found together in the ethanol-responsive turquoise module. Protein synthesis accounts for a large portion of the typical cellular energy expenditure (Lane and Martin, 2010). Multiple turquoise module genes involved in either protein translation (*EIF5* and *MRPL47*) or cellular respiration/mitochondrial function (*MFF, GLRX3, NDUFS3, MRPL47, UQCRC2*, and *TOMM22*) had highly ranked ERHS values. Ethanol alters mitochondrial size and morphology in neurons (Tavares and Paula-Barbosa, 1983), possibly by disruption of mitochondrial fission. The gene *MFF* encodes a protein involved in mitochondrial fission which recruits dynamin-1-like protein (DNM1L; a GTPase that participates in fission), which is also found within the turquoise module. *GLRX3* encodes glutaredoxin 3, an oxidoreductase enzyme that reduces many substrates by a glutathione-dependent mechanism. This gene may be of particular interest, as oxidative stress, especially with ethanol-induced oxidative mitochondrial damage, has been proposed as a major contributor to the neurotoxic effects of the drug (Hoek et al., 2002; Hernández et al., 2016).

The turquoise module also was enriched for genes involved in the ubiquitin/proteasome system. Within the top 25 turquoise genes by ERHS were three components of the 26S proteasome (*PSMD14, PSMC6*, and *PSMB6*), a ubiquitin-conjugating enzyme (*UBE2N*), and a ubiquitin ligase (*RNF138*). Ethanol treatment alters proteasome gene expression and activity in neuroblastoma cells (Caputi et al., 2016). Furthermore, induction of P450 cytochrome 2E1 in the rat liver by chronic ethanol feeding is associated with attenuation of proteasome activity, likely by inhibition of proteasome assembly (Bardag-Gorce et al., 2005), and chronic ethanol-induced inhibition of the proteasome and the resulting epigenetic effects have been proposed as a mechanism contributing to liver cell damage (Bardag-Gorce, 2009).

### Common Themes Across Modules

Analysis of genomic data herein was primarily module-based, but some gene functions appeared across multiple modules, often represented by genes with high ERHS. REVIGO-summarized (see Supplementary Methods) functional enrichment analysis of the top 250 genes by ERHS from the entire study showed enrichment for genes involved in myelination, Golgi vesicle transport, response to drug, mRNA modification, AMPA receptor signaling, GTPase activity, oxidoreductase activity, antigen processing, coated membranes, and excitatory synapses (Table 2, full results in Table S14). While genes involved in myelination, oxidoreductase activity, and glutamate signaling were each enriched in a single module, other gene ontologies such as Golgi vesicle transport/coated membranes, GTPase activity, and mRNA modification were enriched across multiple modules. It has been known that ethanol exposure results in disorganization of the Golgi apparatus (Renau-Piqueras et al., 1985; Romero et al., 2015), a process that likely involves GTPase activity and COPII-mediated vesicular transport (Petrosyan et al., 2015) and can lead to altered lipid metabolism and neurite development (Powrozek and Olson, 2012); all processes strongly implicated by our study in the biological effects of chronic ethanol.

**Table 2:** Top 10 functional enrichment categories within the top 250 genes by ERHS from the entire study, as summarized by REVIGO from the ToppFun results.

**Ontology ID**	**Ontology type**	**Description**	***p*-value**	**Dispensability**	**Frequency**	**Uniqueness**
GO:0043209	Cellular component	Myelin sheath	4.80E-06	0	0.01%	0.929
GO:0048193	Biological process	Golgi vesicle transport	5.56E-05	0	0.04%	0.736
GO:2001025	Biological process	Positive regulation of response to drug	4.14E-04	0	0.00%	0.75
GO:0016556	Biological process	mRNA modification	8.13E-04	0	0.02%	0.817
GO:0004971	Molecular function	AMPA selective glutamate receptor activity	8.17E-04	0	0.00%	0.939
GO:0031267	Molecular function	Small GTPase binding	9.25E-04	0	0.03%	0.854
GO:0018660	Molecular function	4-Hydroxyphenylacetate, NADH: oxygen oxidoreductase (3-hydroxylating) activity	2.79E-03	0	0.00%	0.335
GO:0002474	Biological process	Antigen processing and presentation of peptide antigen via MHC class I	5.15E-03	0	0.06%	0.971
GO:0048475	Cellular component	Coated membrane	5.18E-03	0	0.10%	0.942
GO:0060076	Cellular component	Excitatory synapse	9.83E-03	0	0.00%	0.854

When looking at functional enrichments across all ethanol-related modules, it is easy to see how ethanol can alter the expression of thousands of genes in the brain. Ethanol-related modules were enriched for chromatin modification, transcription factors, helicases, RNA polymerases, RNA splicing, translation regulators, ribosomal proteins, Golgi apparatus function, and ubiquitin/proteasome function. This suggests that chronic ethanol may alter gene expression at any point from pre-transcriptional regulation to protein degradation, in agreement with previous findings in ethanol-treated neuroprogenitor cells (Garic et al., 2014).

### Ethanol-Related Modules Conserved Between Primates and Rodents

Cross-species analysis of expression data resulted in the discovery of several ethanol-related gene networks that were conserved between primates and rodents. Not only does this discovery help to focus on genes and networks that are central to ethanol's biological effects in mammals (and thus have high therapeutic potential), but it also speaks to the translational nature of genetic/genomic studies of alcohol abuse in rodents.

For the most part, gene functions enriched within meta-modules mirrored those of modules discovered in the monkey-alone analysis. Indeed, every meta-module had a correlate monkey module. Looking in the other direction, only half of the monkey modules had a correlate meta-module; the other half of the monkey modules were presumably constructed based on gene-gene correlations that were strong in the monkey but not in the mouse. Within the overlap of monkey modules with meta-modules, several functional enrichments that were strong in the monkey-alone analysis were present, including myelination, synaptic transmission, RNA processing, translation, and cellular respiration. However, several other functional categories emerged within the overlaps that were less evident in the monkey-alone analysis, such as antigen processing, circadian rhythms, steroid receptor binding, kinase inhibitors, and protein localization to nucleus.

The cross-species analysis is remarkable given that exact functional anatomical correlates between rodent and primate cortex are not without controversy in the literature (Uylings et al., 2003). We did make an effort to restrict our genomic studies to highly related areas of medial PFC in the monkey and mouse brain, but admit that evolutionary differences and technical limitations on dissections may have affected our cross-species analysis to a degree.

### Monkey Module Overlap With Gene Sets From Other Ethanol Genomic Studies

Several monkey modules showed significant overlap with ethanol-related gene sets from genomic studies by different investigators that have been deposited in the GeneWeaver bioinformatics resource. For simplicity, Table S8 shows overlaps with gene sets from a selection of studies that illustrates the cross-species relevance of our findings. These gene sets include: acute ethanol-regulated genes in the PFC of mice (Wolen et al., 2012), chronic ethanol-regulated genes in the PFC of mice (van der Vaart et al., 2017), and genes differentially expressed in the frontal cortex (Lewohl et al., 2000; Liu et al., 2006), hippocampus (McClintick et al., 2013; Farris et al., 2015), and amygdala (Ponomarev et al., 2012) of human alcoholics vs. control subjects.

The degree of overlap of our monkey modules with data from these other studies was quite striking (Figure S16). For the most part, functions enriched within the overlapping genes mirrored those found within the monkey modules themselves: myelination, synaptic transmission, chromatin modification, Golgi function, translation, respiration, RNA processing. However, the GeneWeaver analysis highlighted some ontologies less-significantly enriched in the monkey modules, including cell cycle, kinase activity, transcription factor activity, calcium regulation, and G-protein signaling. Finding significant overlap with such a variety of ethanol-related gene sets validates aspects of our findings not only across species, but also across brain areas, investigators, array platforms, and ethanol treatment paradigms.

## Conclusion

Our extensive genomic analysis of expression networks in medial prefrontal cortex of rhesus macaques has identified networks strongly over-represented for coherent biological functions relevant to brain plasticity and toxicity that might occur with chronic ethanol consumption. Many of our findings appear robust in that they replicate aspects of prior genomic or molecular studies on ethanol in macaques or other species. Furthermore, our cross-species analysis with a chronic ethanol exposure model in mice also shows extensive replication of networks. In particular, we identify a myelin-related gene network as having striking connectivity changes following ethanol consumption in rhesus and a similar network is also seen in the mouse. Hub genes in these and other networks identified here may represent key nodes for understanding mechanisms of brain adaptation to ethanol and could possibly lead to new therapeutic targets for AUD. Additionally, our studies suggest a dichotomy between gene networks involved in ethanol consumption and those enriched in genes actually regulated by ethanol exposure. This finding may be of fundamental importance in understanding the overall role of brain gene expression in the neurobiology of ethanol consumption vs. toxicity.

## Data Availability

The datasets generated for this study can be found in the Gene expression data uploaded to the Gene Expression Omnibus (accession number GSE134546) and full expression data are included in Supplementary Tables herein, module data uploaded to GeneWeaver.

## Ethics Statement

All primate procedures were conducted in accordance with the NIH and the Guide for the Care and Use of Laboratory Animals and protocols approved by the Oregon National Primate Research Center IACUC. All mouse studies were conducted in an AALAC-accredited animal facility and protocols approved by the Institutional Animal Care and Use Committee of MUSC. All experimental and animal care procedures met guidelines outlined in the NIH Guide for the Care and Use of Laboratory Animals.

## Author Contributions

JB ran all network analyses and other data analyses, contributed to experimental design, generated figures and tables, and authored manuscript. MS analyzed microarrays on mouse samples, assisted in network analyses, and contributed to experimental design. SF contributed preliminary data leading to the study. CD ran microarrays on monkey brain samples. ML generated ethanol vapor-exposed mice and collected mouse drinking data. HB generated ethanol vapor-exposed mice and microarrays from mouse brain tissue. KG generated chronic ethanol-drinking monkeys and collected monkey phenotype data. MM contributed to experimental design and interpretation of all data analyses and co-authored/edited manuscript.

### Conflict of Interest Statement

The authors declare that the research was conducted in the absence of any commercial or financial relationships that could be construed as a potential conflict of interest.

## Footnotes

^1^http://matrr.com

^2^http://www.r-project.org

^3^http://www.unmc.edu/rhesusgenechip

^4^https://www.ncbi.nlm.nih.gov/geo

^5^https://www.geneweaver.org

^6^www.GeneMania.org

^7^www.geneweaver.org
