# Supplementary material for: Cross-Species Co-analysis of Prefrontal Cortex Chronic Ethanol Transcriptome Responses in Mice and Monkeys
Source: Front Mol Neurosci. 2019 Aug 13;12:197. doi: 10.3389/fnmol.2019.00197 (PMC6701453; doi:10.3389/fnmol.2019.00197)
Supplement: Supplementary file 15 [file Table_15.DOCX]

**Supplementary Figure Legends**

**Figure S1** – Principal component analyses showing data structure before (left) and after (right) ComBat normalization. Plots show identical data, color coded by microarray processing batch (**A, B**), MATRR cohort (**C, D**), and treatment group (**E, F**). Note that ComBat removed the batch effects of microarray processing batch and MATRR cohort, while still leaving some noticeable difference between treatment groups.

**Figure S2** – Multi-dimensional scaling plots for deep-split values 0-3 (**A-D**, respectively).

**Figure S3** – Hierarchical clustering dendrogram of all probesets (top) and the assignment of branches of probesets into color-coded modules under deep-split values of 0-3 (bottom).

**Figure S4** – Histogram of ERHS values for all probesets. 31,479 probesets were divided into 100 bins.

**Figure S5** – Relationship of monkey and mouse expression and connectivity values for homologous gene pairs. A strong positive correlation was seen for ranked expression of homologous gene pairs (left), but not for ranked connectivity (right).

**Figure S6** – Q-Q plot of topological overlap values for monkey and mouse homologous gene pairs before (black) and after (red) scaling. Following scaling, monkey and mouse TO data were highly similar. The blue line has a slope of 1.

**Figure S7** – Phenotype-phenotype correlations. Positive correlations are colored red, and negative correlations are colored green. At each intersection, the top number represents the Pearson correlation R-value, and the bottom number in parentheses is the p-value.

**Figure S8** – Green module characteristics: chromatin modification. (**A**) Select functional enrichment categories from ToppFun are shown which pertain to chromatin modification. (**B**) Circle plot of blue module genes from the “chromatin modification” ontology that were also ethanol-regulated by LIMMA (p<0.01) showed a clear decrease in connectivity in the ethanol-treated animals. (**C**) A network diagram from GeneMania supports the finding that the chromatin modification genes in B are highly interconnected, using data from the literature. This network contains several potential hub genes. The arrangement of nodes was modified to better display the number of connections to each query gene (black). Genes added to the network based on connections from the literature are shown in grey.

**Figure S9** – Steelblue module characteristics. (**A**) The top 10 functional enrichments from the REVIGO summary of ToppFun analysis are shown. Many pertain to circadian rhythms. (**B**) Circle plots showing the whole steelblue module. Note that the circadian clock genes (CRY2, PER2, PER1) are hub genes in the control animals but are far less connected in the ethanol-treated animals. (**C**) A network diagram from GeneMania supports the finding that circadian clock genes (right center) represent important hubs within this network, using data from the literature. Query genes from the steelblue module are shown in black, and genes added to the network based on connections from the literature are shown in grey. (**D**) Scatterplot of steelblue module membership vs. correlation to the blood ethanol concentration phenotype (gene significance) for each probeset in the steelblue module. Note the significant positive correlation.

**Figure S10** – Blue module characteristics. (**A, B**) REVIGO summaries of functional enrichments are shown for the whole module and for the ethanol-responsive genes in the blue module, respectively. (**C**) Positive correlation of blue module gene significance of ethanol regulation by LIMMA and significance of blue module membership.

**Figure S11** - Turquoise module characteristics. (**A, B**) REVIGO summaries of functional enrichments are shown for the whole module and for the ethanol-responsive genes in the turquoise module, respectively. (**C**) Positive correlation of turquoise module gene significance of ethanol regulation by LIMMA and significance of turquoise module membership.

**Figure S12** – Pink module characteristics. (**A**) The top 10 functional enrichments from the REVIGO summary of ToppFun analysis are shown. Various functions are represented. (**B**) Circle plot of the top 50 pink module genes by ERHS showing reorganization of connectivity in ethanol-treated animals. (**C**) A network diagram from GeneMania supports the finding that the top ERHS genes within the pink module are highly interconnected, using data from the literature. Genes added to the network based on connections from the literature are shown in grey. (**D**) Scatterplot of the negative log of pink module membership p-value vs. negative log of LIMMA p-value for each probeset in the pink module. Note the highly significant positive correlation.

**Figure S13** – Heatmap showing correlations of each meta-module to each monkey phenotype. The heatmap color scheme is shown at right, with positive correlations being red and negative correlations being green. At each intersection, the top number represents the Pearson correlation R-value, and the bottom number in parentheses is the p-value.

**Figure S14** – Heatmap showing correlations of each meta-module to each mouse phenotype. The heatmap color scheme is shown at right, with positive correlations being red and negative correlations being green. At each intersection, the top number represents the Pearson correlation R value, and the bottom number in parentheses is the p-value.

**Figure S15** – Heatmap showing overlap of monkey modules with meta-modules. The heatmap color scheme is shown at right, with significant p-values of the hypergeometric test shown in red. Numbers at each intersection represent the number of overlapping genes.

**Figure S16** – Heatmap showing overlap of the five monkey modules that had correlate meta-modules (X-axis) with seven ethanol-related gene sets from GeneWeaver discovered in different species and brain areas (Y-axis). The heatmap color scheme is shown at right, with significant p-values of the Fisher’s Exact Test shown in red. The number at each intersection represents the number of overlapping genes.

**Supplementary Table Legends**

**Table S1** – Detailed descriptions of monkey phenotypes used for correlation to WGCNA module eigengenes, as well as the phenotype data for each monkey.

**Table S2** – Spreadsheet containing the full ToppFun functional enrichment results of all monkey modules.

**Table S3** – Spreadsheet containing RMA data and LIMMA results for each monkey probeset.

**Table S4 –** Spreadsheet containing connectivity and module membership data for each monkey probeset.

**Table S5** – Spreadsheet containing Pearson correlation R-values and p-values for each probeset to each phenotype. Columns beginning with GS are R-values, and columns beginning with p.GS are p-values.

**Table S6** – Table showing the size of all monkey modules produced by WGCNA, as well as the enrichment with ethanol-regulated genes as determined by LIMMA, the module eigengene correlation to the ethanol intake phenotype, and whether the module has a correlate meta-module from the monkey-mouse consensus module analysis. Significant enrichments and correlations are highlighted. Modules that both meet one of the criteria for ethanol-relatedness and have a correlate meta-module are shown in bold with an asterisk.

**Table S7** – The topological overlap (TO) of each module was compared to the mean TO of 100 permutations of randomly generated modules of the same size. All modules generated by WGCNA had very significantly different TO than the random modules, with the exception of the grey module, which is a catch-all of genes that do not belong in another module.

**Table S8** – Overlaps of select monkey modules that had correlate meta-modules with seven ethanol-related gene sets from GeneWeaver discovered in different species and brain areas. P-values for Fisher’s exact test are provided, as are author-generated summaries of functional enrichments within each overlap.

**Table S9** – Cell type enrichment results. 14 out of 30 modules showed significant enrichment for a particular cell type. P-values for the hypergeometric test are in the rightmost column.

**Table S10 -** Spreadsheet with meta-module assignments, RMA values, and meta-module membership values from the monkey-mouse consensus module analysis.

**Table S11** – Spreadsheet with complete ToppFun functional enrichment results for all meta-modules.

**Table S12** – Monkey module – meta-module overlaps. P-values for the hypergeometric test are provided.

**Table S13** – Meta-module sizes and correlations to monkey and mouse drinking behaviors. Significant correlations are highlighted.

**Table S14** – Spreadsheet with complete ToppFun functional enrichment results for the top 250 genes by ERHS in the entire study.
