# Supplementary material for: Cross-Species Co-analysis of Prefrontal Cortex Chronic Ethanol Transcriptome Responses in Mice and Monkeys
Source: Front Mol Neurosci. 2019 Aug 13;12:197. doi: 10.3389/fnmol.2019.00197 (PMC6701453; doi:10.3389/fnmol.2019.00197)
Supplement: Supplementary file 16 [file Data_Sheet_1.docx]

**Supplementary Methods**

**Additional details on monkey necropsy**

After one year of open access to ethanol, animals were necropsied. Within four hours of last access to ethanol, animals were sedated with ketamine (15 mg/kg i.m.), prepped for surgery, and anesthetized with sodium pentobarbital (20-35 mg/kg i.v.). Craniotomy was followed by transcardial perfusion with oxygenated buffer solution, rapid brain extraction and dissection, and flash-freezing of tissue samples. Tissue samples were deposited into the Monkey Alcohol Tissue Research Resource, a tissue bank created with the goal of distributing alcohol-related tissue samples and relevant bioinformatic data to the alcohol research community. The animals used in this study comprised rhesus cohorts 4, 5, 7a, and 7b of the MATRR. A detailed diagram of the experimental timeline for each cohort can be found at <http://matrr.com>.

**Additional details on mouse subjects**

Prior to CIE, drinking groups underwent 6 weeks of 2-bottle choice drinking (15% ethanol v/v in water; 2 h/d) to establish baseline drinking levels. Ethanol and water intake for each mouse were measured daily. Following baseline drinking, mice were placed in plexiglass inhalation chambers (60x36x60 cm) 16 hours/day for 4 days. Mice then underwent a 72-hour period of abstinence from ethanol, after which the mice in the drinking groups were given 2-bottle choice drinking, 2 hours per day for 5 days. Four consecutive cycles of CIE-abstinence-drinking were performed prior to sacrifice. Animals were sacrificed and brain tissue rapidly dissected at 24-hours after the 4^th^ day of ethanol/water drinking day, a time when the animals would have gotten their 5^th^ day of ethanol/water consumption. Thus, all mice had been withdrawn from ethanol for 7 days (CIE Non-Drinking) or 24-hours (CIE Drinking or Air Drinking groups) at the time of sacrifice. Coronal slices (1 mm) were punch dissected (1.25 mm dia) in medial PFC area and included tissue from prelimbic, infralimbic and anterior cingulate cortex due to the thickness of the slice and diameter of the punch biopsy (see: <http://atlas.brain-map.org/atlas?atlas=1&plate=100960372#atlas=1&plate=100960365&resolution=9.31&x=4167.999945746527&y=3510.0012885199653&zoom=-3&z=7&structure=211>). For WGCNA meta-analysis of phenotype/meta-module correlations, the CIE Drinking and Air Drinking animals were considered the “ethanol-drinking” group, while CIE Non-Drinking and Air Non-Drinking animals were used as the non-drinking group. Full experimental details of the mouse CIE/drinking genomics manuscript (submitted for publication) can be found in preprint form at BioRxiv (<https://www.biorxiv.org> MS ID#: BIORXIV/2019/688267)

**Bioinformatics**

Monkey modules identified with WGCNA were examined for functional enrichment using the ToppFun tool in the online ToppGene Suite (https://toppgene.cchmc.org/). Functional enrichment analysis was performed with p-value cutoff of 0.01 within the ontological categories Molecular Function, Biological Process, Cellular Component, Human Phenotype, Mouse Phenotype, Pathway, Gene Family, and Disease, focusing on ontologies containing between 3 and 1,000 genes. Full ToppFun results are found in Table S2. For summary display in figures, ToppFun results were processed to decrease redundant categories using REVIGO (http://revigo.irb.hr/), which only accepts GO categories Molecular Function, Biological Process, and Cellular Component. REVIGO settings were left at default, except for output list size, which was set to small (0.5).

Modules or selections from modules were validated (in part) by processing gene lists with GeneMania (http://www.genemania.org/), which uses data from the literature to construct a network from the query genes provided. GeneMania networks were generated with the following changes to the default settings. Co-localization and Shared Protein Domains connections were turned off. Under Co-expression, Alter-Stephan-2011 was added. Under Genetic Interactions, Lin-Smith-2010 was removed. In cases where network connections were so abundant as to obstruct interpretation, query gene nodes were manually rearranged into a circular shape to discern roughly how many connections each node had.

All modules from this publication were uploaded to GeneWeaver.org, a community-built and curated data archive of gene sets and gene networks. This website allows for comparison of gene sets across studies, species, tissues, and experimental platforms, which can both provide validation of genomic findings and lead to discovery of novel relationships between gene networks. Modules were examined for overlap with gene networks from other studies, using the Jaccard Overlap tool within GeneWeaver. Overlaps between modules/gene sets were tested for significance in R using Fisher’s Exact Test (p≤0.05) and a genome size of 26,000 genes.

**Cell Type Enrichment Analysis**

Cell type enrichment analysis was performed on WGCNA modules using the userListEnrichment function within the WGCNA package for R (Miller et al. 2011), which employs a hypergeometric test. The cell type-specific gene expression data used in this analysis was compiled from two previous studies (Cahoy et al. 2008, Zeisel et al. 2015). The data taken from the Cahoy study were filtered for genes having 3-fold or higher enrichment in each cell type. Genes enriched in all neuronal subtypes from the Zeisel study were combined into a single neuronal category, along with the neuron-enriched genes from the Cahoy study. This resulted in a cell type enrichment dataset comprising 5,329 unique genes divided into the following categories: neuronal, oligodendrocytic, astrocytic, microglial, ependymal, vascular epithelial, and vascular mural.

**Collation of Homologous Genes for Cross-Species Analysis**

In order to co-analyze monkey and mouse expression data, a list of homologous monkey-mouse gene pairs had to be collated. The list of annotated rhesus macaque genes included on the Affymetrix GeneChip Rhesus Macaque Genome Arrays was queried to find mouse homologs using data from Ensembl BioMart (http://www.ensembl.org/biomart) and NCBI Homologene (http://www.ncbi.nlm.nih.gov/homologene). In cases where a gene was represented by multiple microarray probesets, data from each species were collapsed so that each homologous gene pair contained expression data from only one microarray probeset per species. Collapsing of expression data was performed using the collapseRows function in the WGCNA package (Miller et al. 2011), by the MaxMean method, which chooses the probeset for each gene with the highest mean expression across samples. To avoid spurious module construction due to genes with very low expression, an abundance filter was applied to remove genes with RMA values less than 3.75 in ≥80% of monkey samples. Among the remaining homologous gene pairs, less than 1% had low expression (by the same criteria as monkey data) in the mouse data.
