# Supplementary figures and images for: Cross-Species Co-analysis of Prefrontal Cortex Chronic Ethanol Transcriptome Responses in Mice and Monkeys
Source: Front Mol Neurosci. 2019 Aug 13;12:197. doi: 10.3389/fnmol.2019.00197 (PMC6701453; doi:10.3389/fnmol.2019.00197)

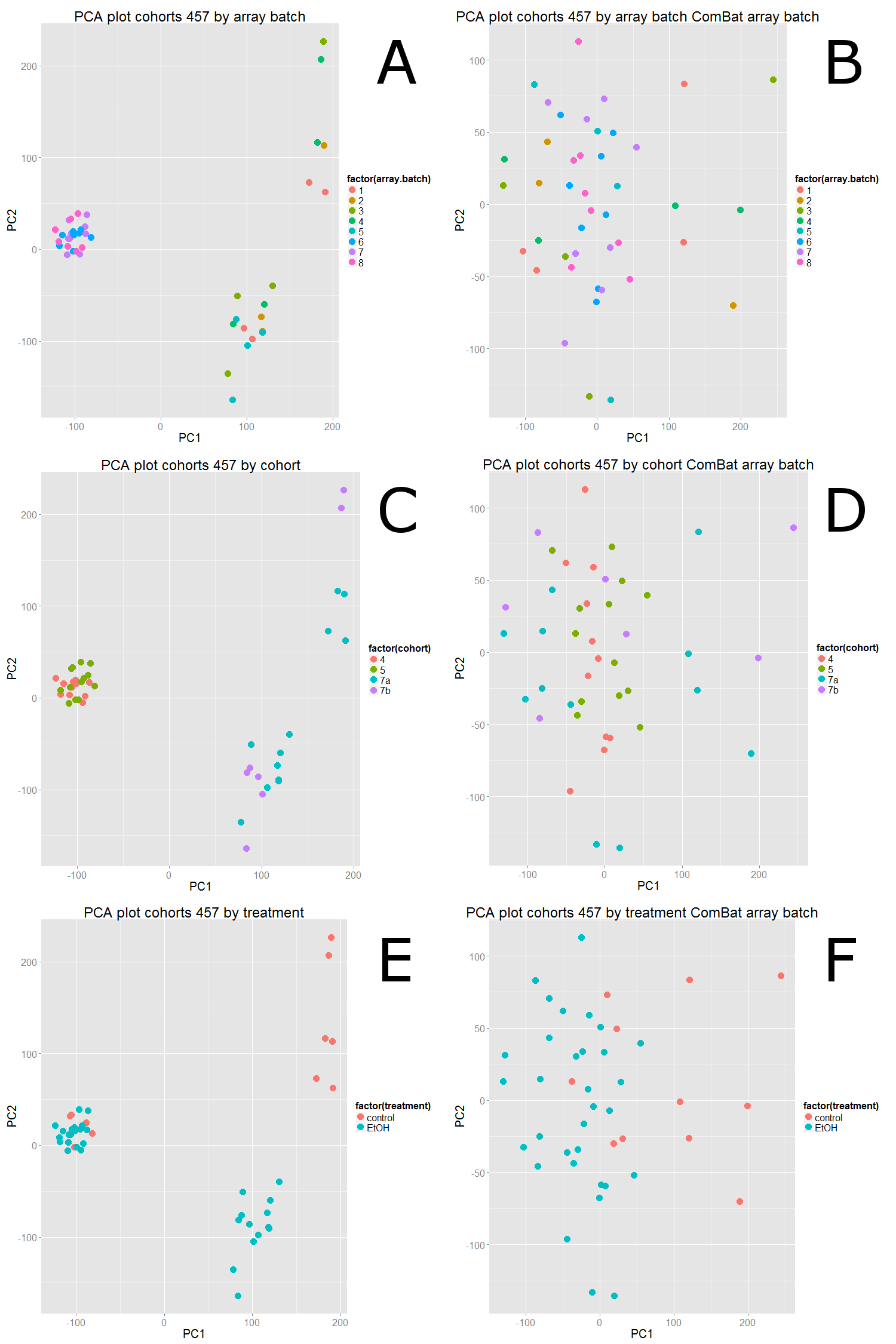

Supplement: Supplementary file 17 [file Data_Sheet_2.zip › Image 1.TIF]

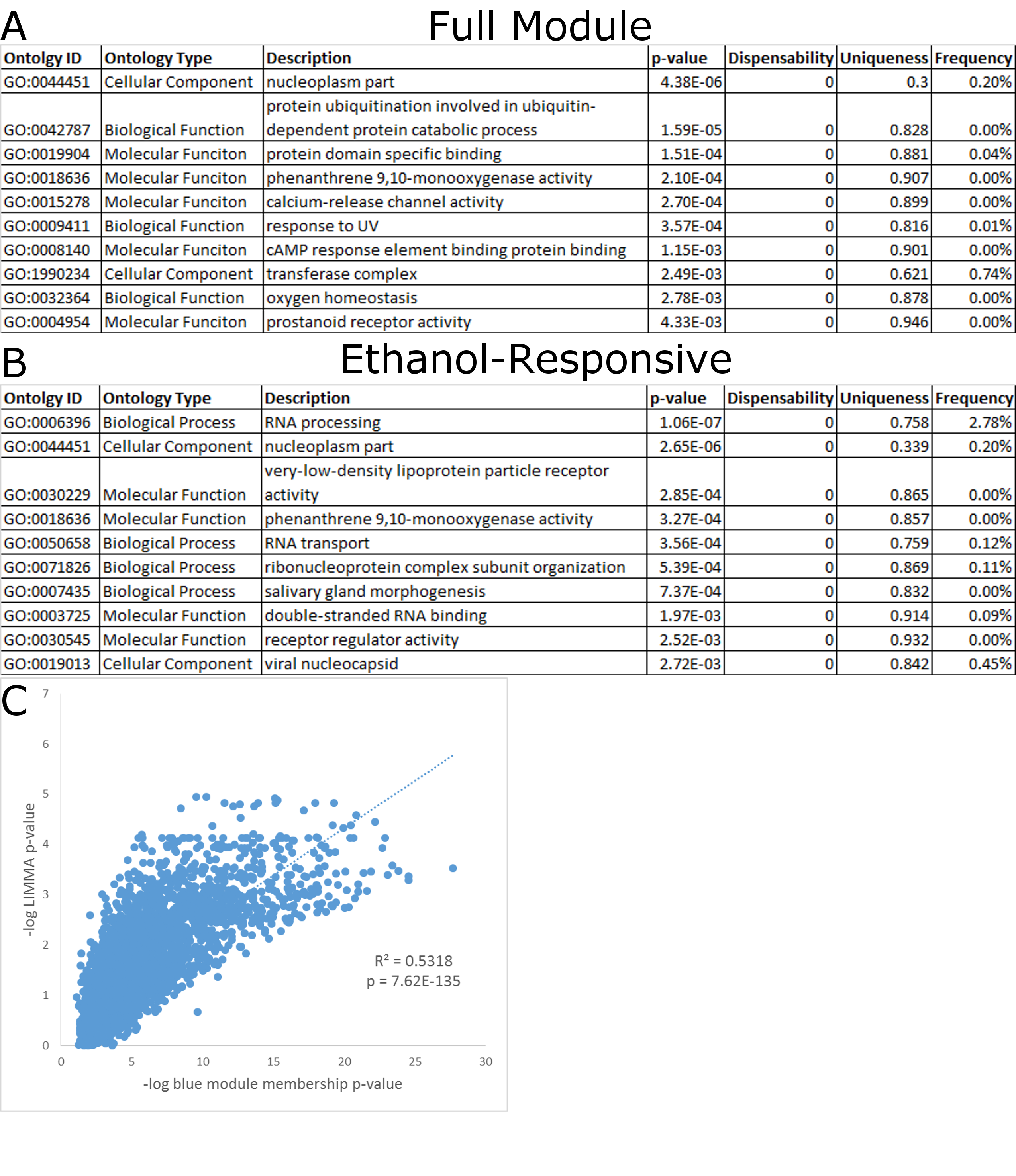

Supplement: Supplementary file 17 [file Data_Sheet_2.zip › Image 10.TIFF]

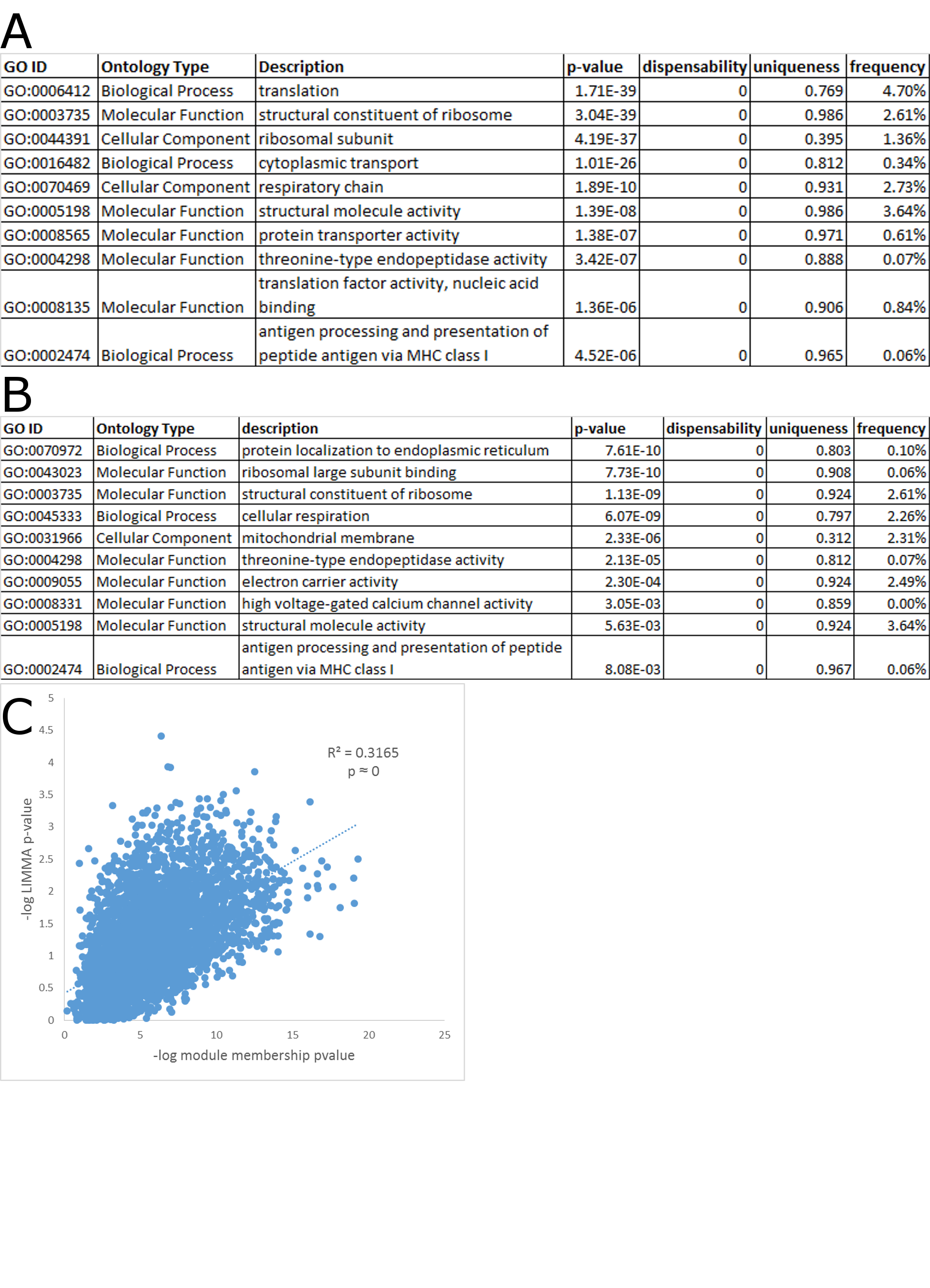

Supplement: Supplementary file 17 [file Data_Sheet_2.zip › Image 11.TIF]

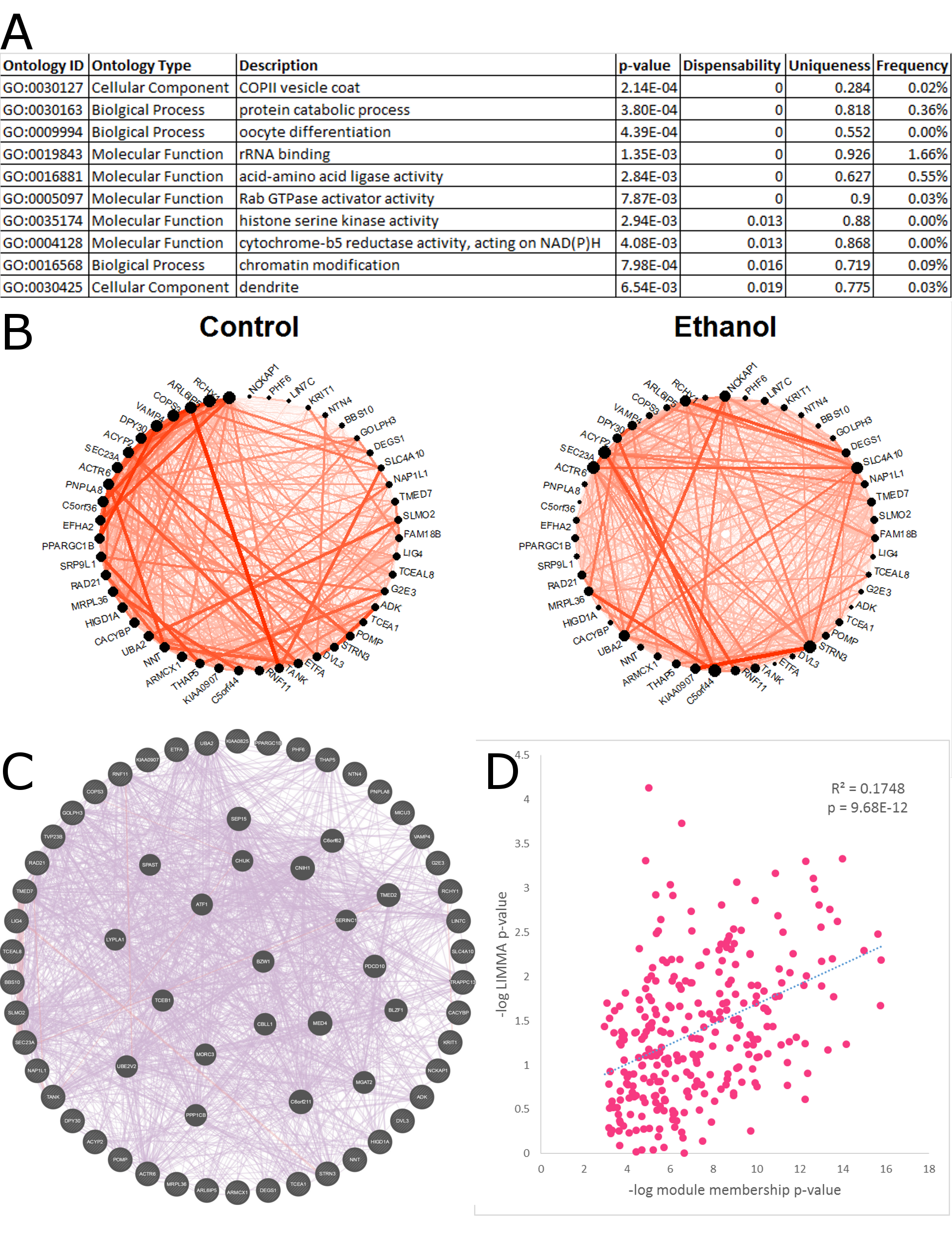

Supplement: Supplementary file 17 [file Data_Sheet_2.zip › Image 12.TIF]

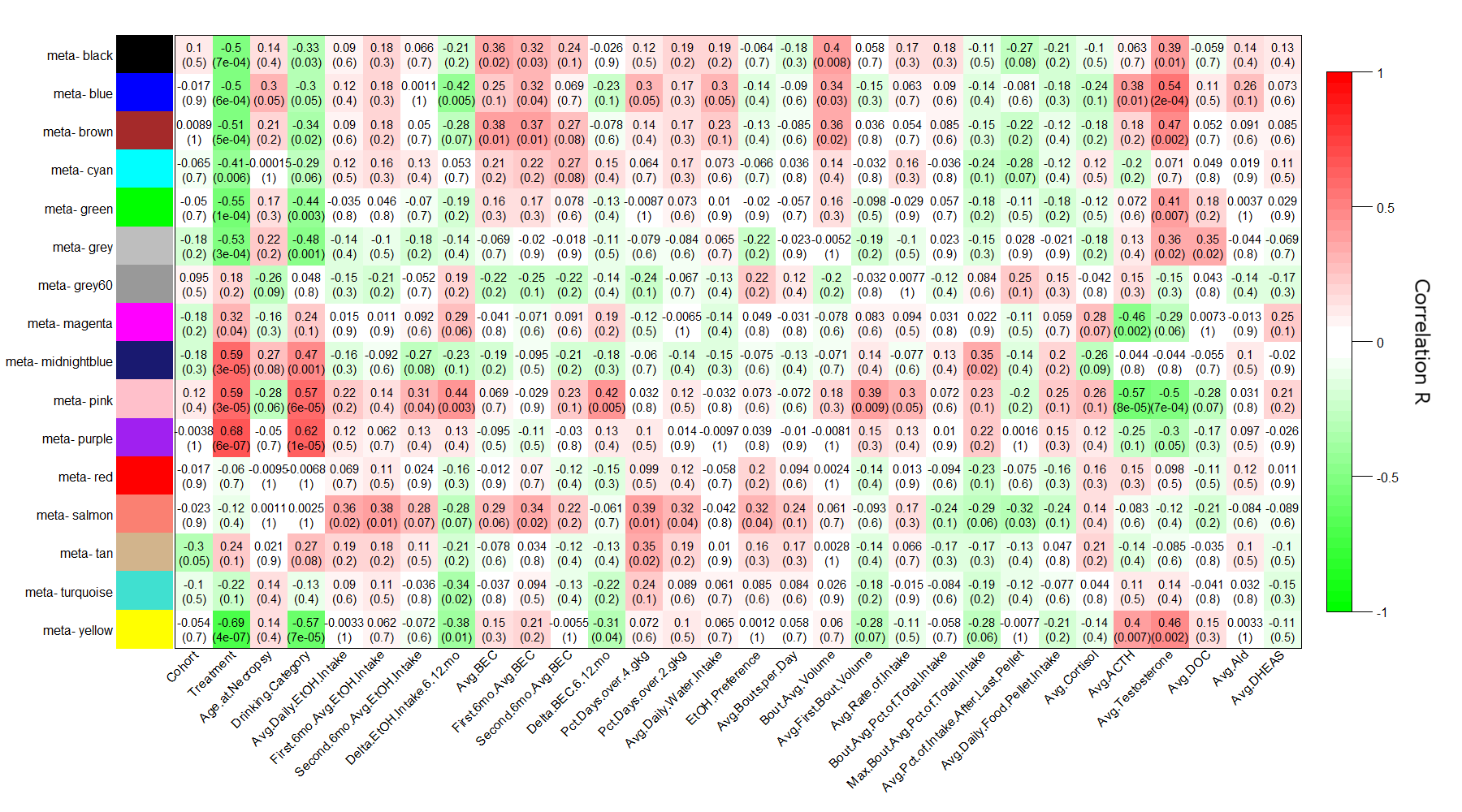

Supplement: Supplementary file 17 [file Data_Sheet_2.zip › Image 13.TIFF]

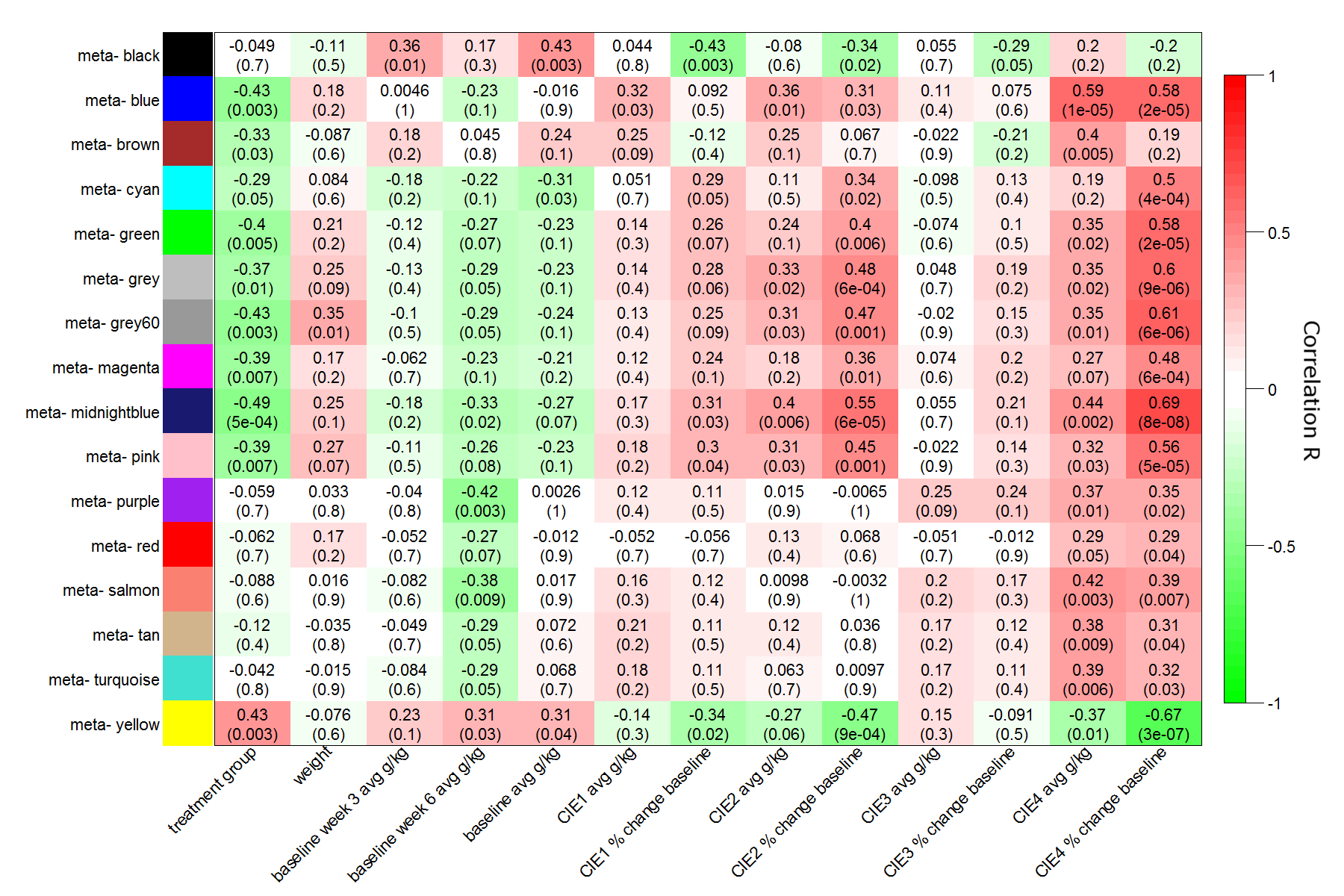

Supplement: Supplementary file 17 [file Data_Sheet_2.zip › Image 14.TIFF]

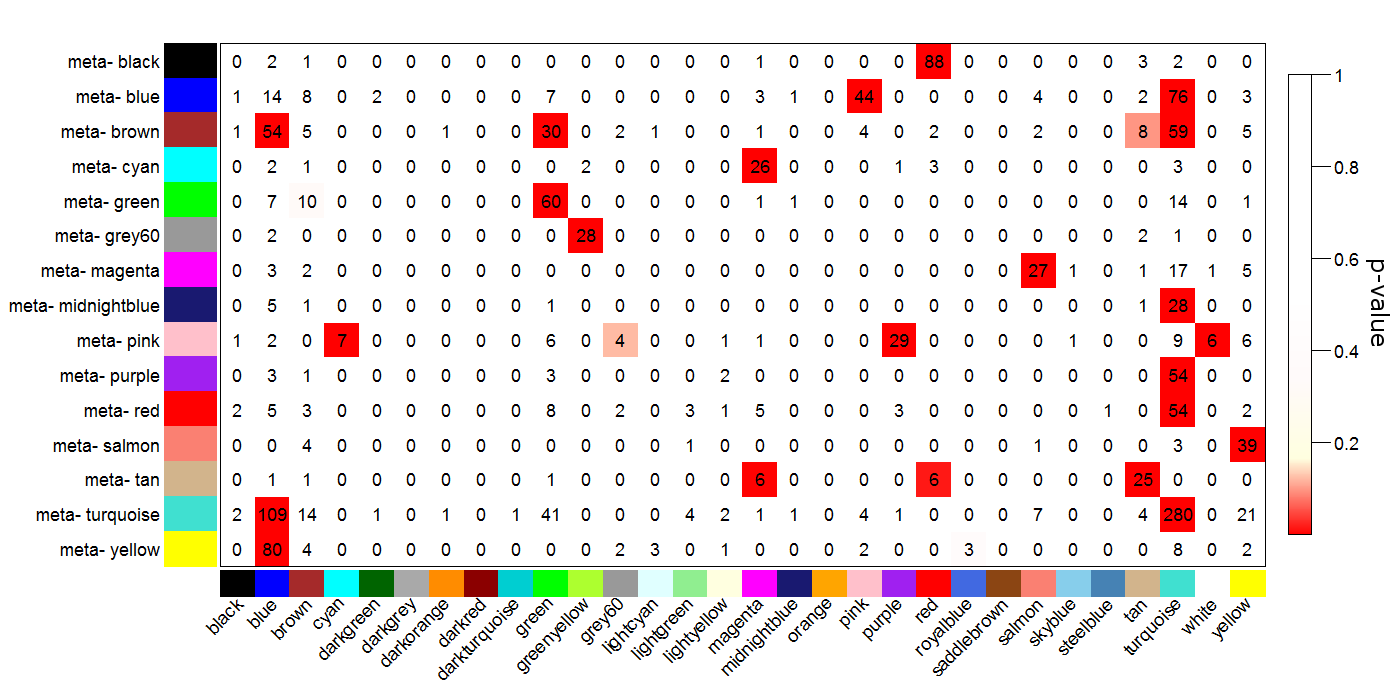

Supplement: Supplementary file 17 [file Data_Sheet_2.zip › Image 15.TIFF]

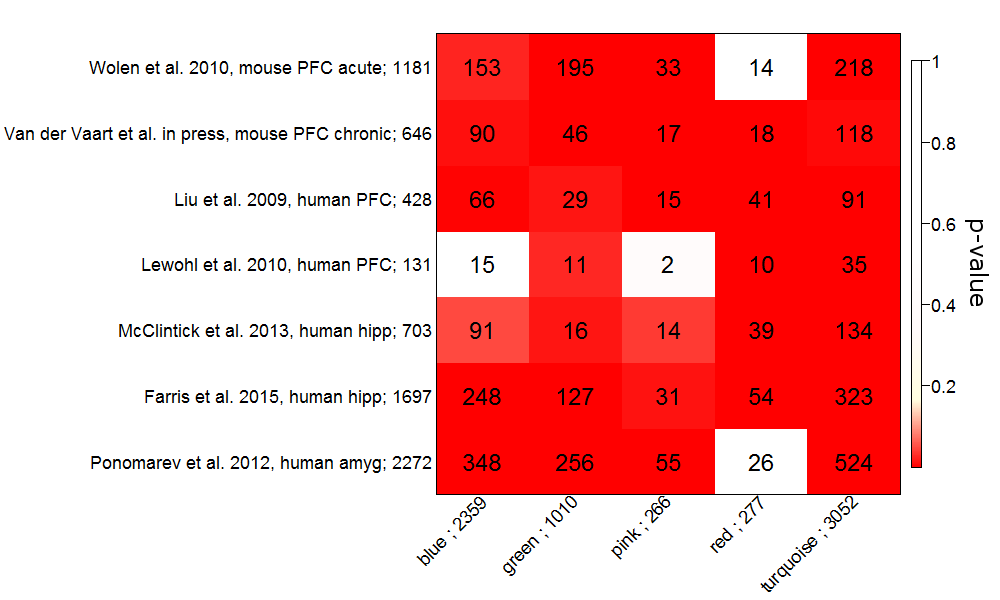

Supplement: Supplementary file 17 [file Data_Sheet_2.zip › Image 16.TIFF]

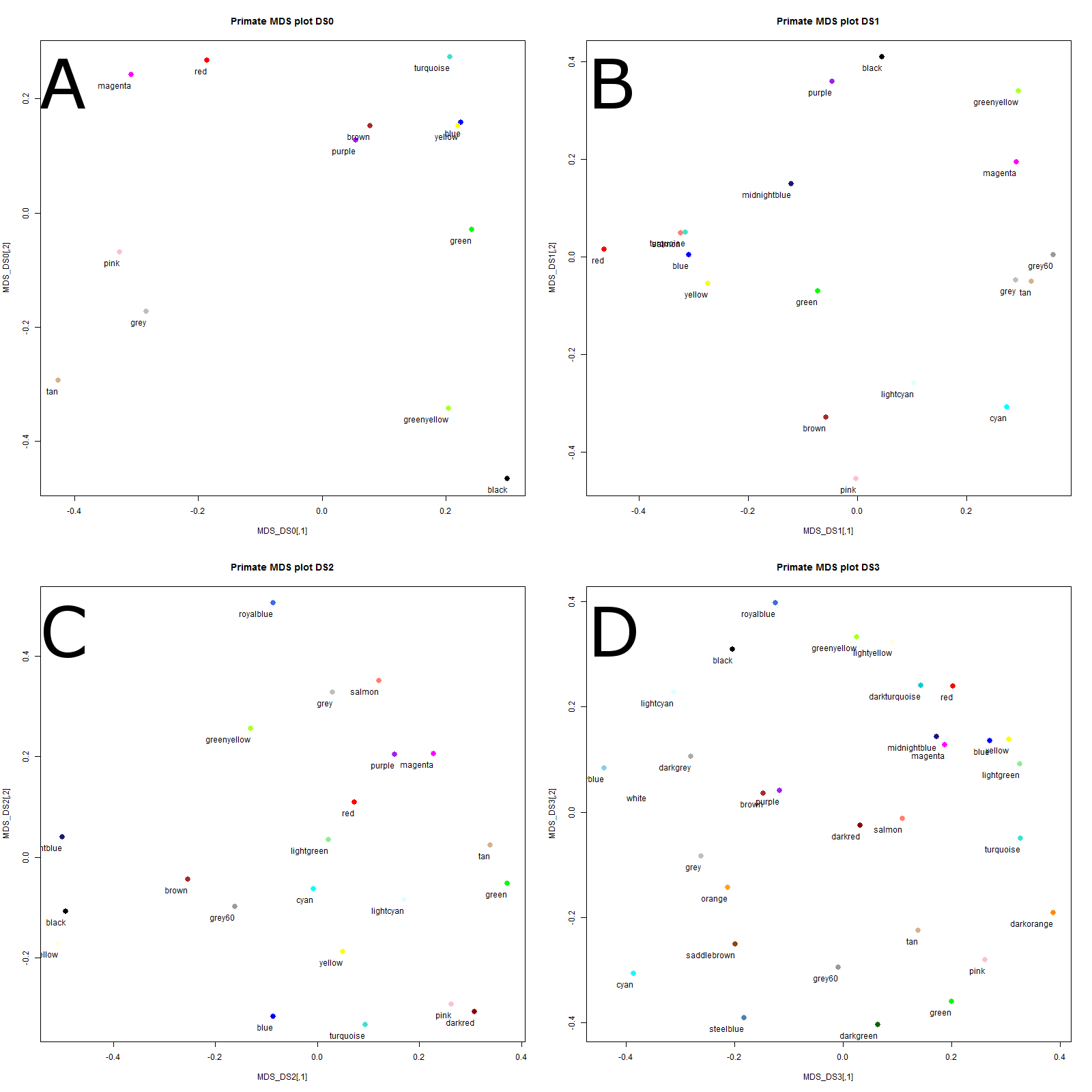

Supplement: Supplementary file 17 [file Data_Sheet_2.zip › Image 2.TIF]

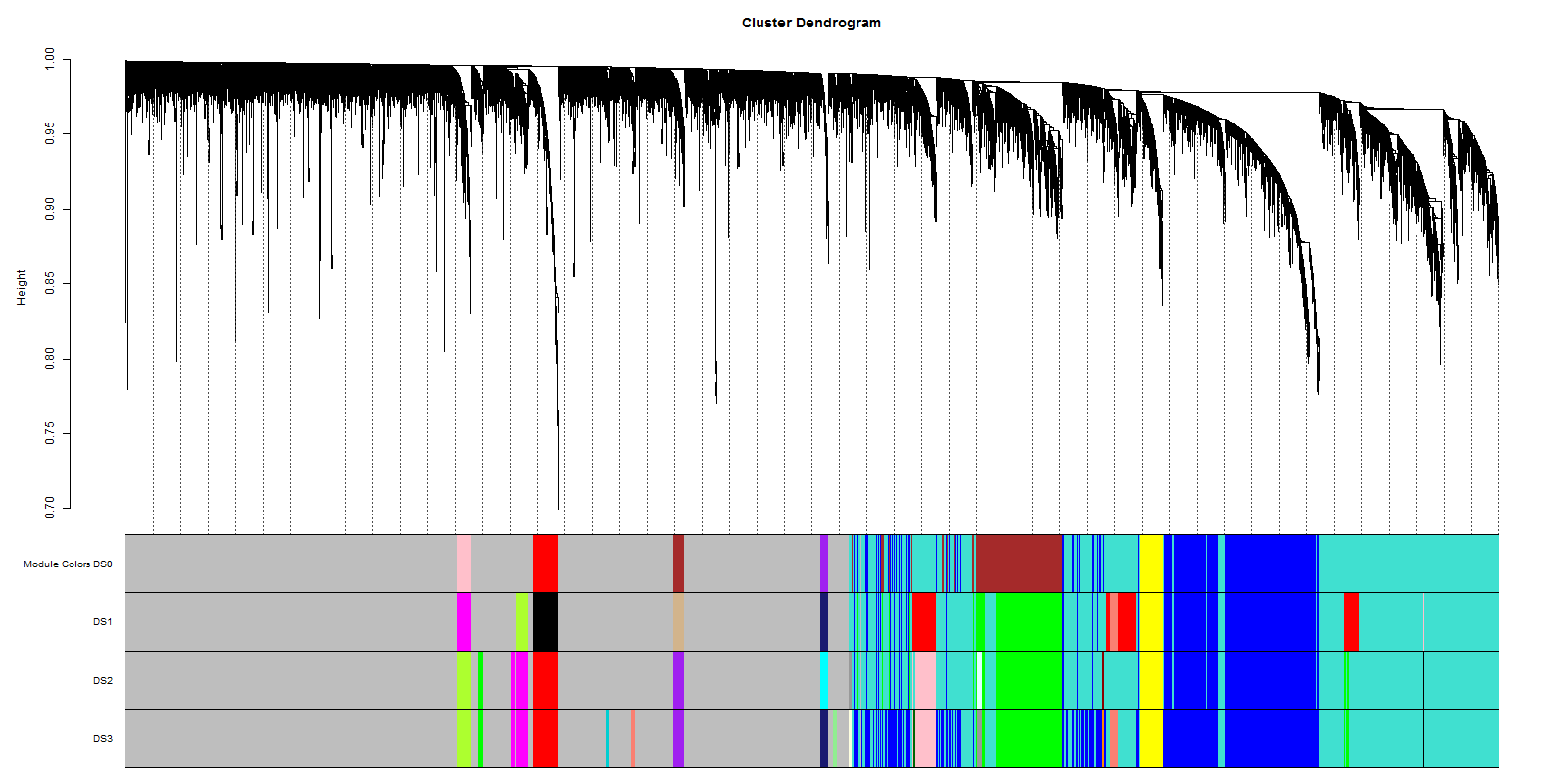

Supplement: Supplementary file 17 [file Data_Sheet_2.zip › Image 3.TIFF]

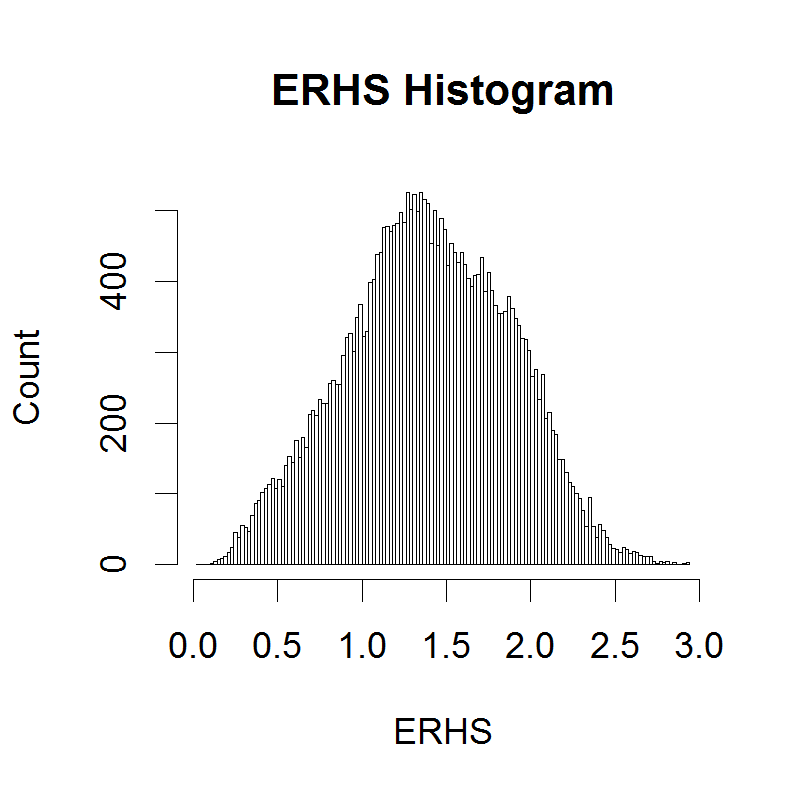

Supplement: Supplementary file 17 [file Data_Sheet_2.zip › Image 4.TIFF]

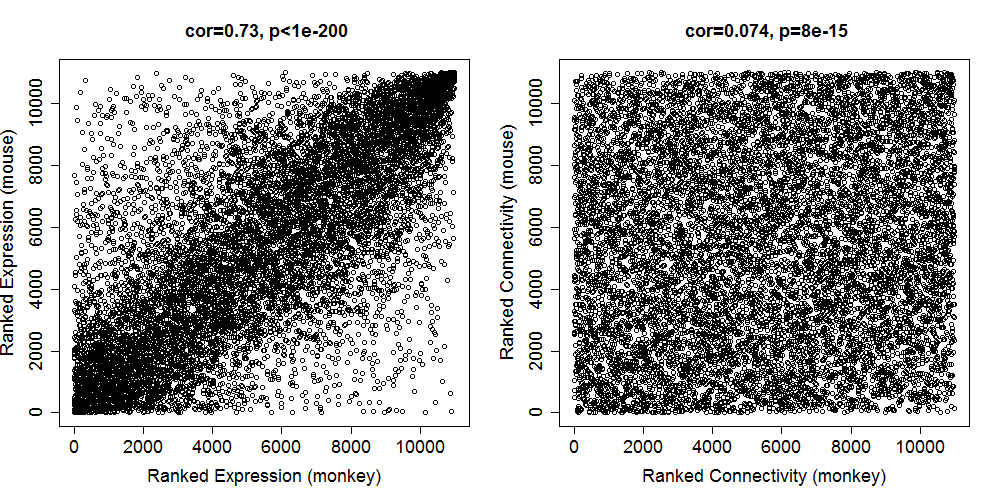

Supplement: Supplementary file 17 [file Data_Sheet_2.zip › Image 5.TIFF]

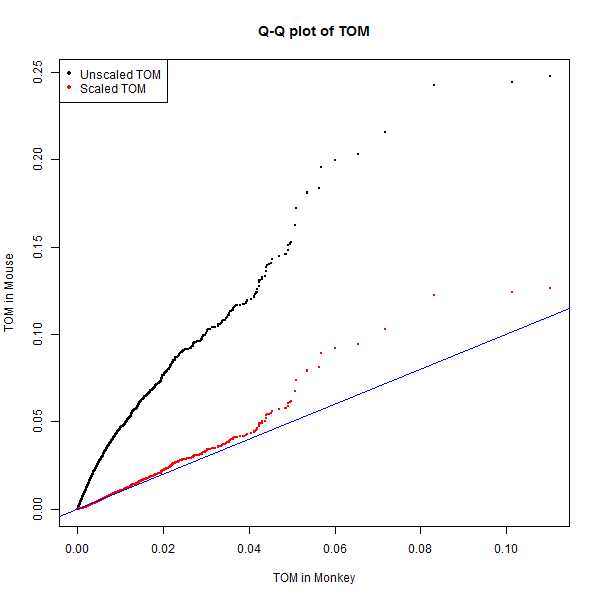

Supplement: Supplementary file 17 [file Data_Sheet_2.zip › Image 6.TIFF]

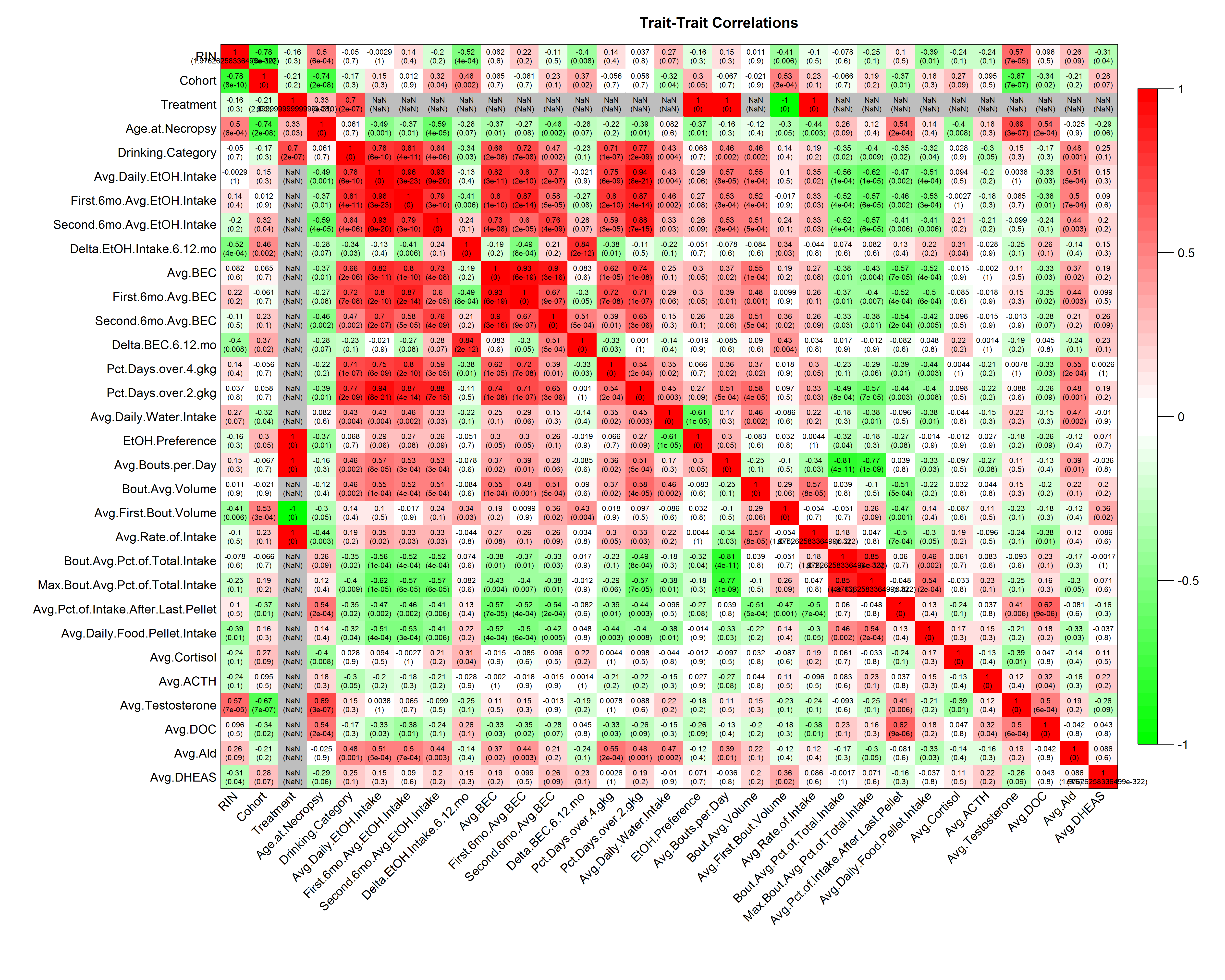

Supplement: Supplementary file 17 [file Data_Sheet_2.zip › Image 7.TIFF]

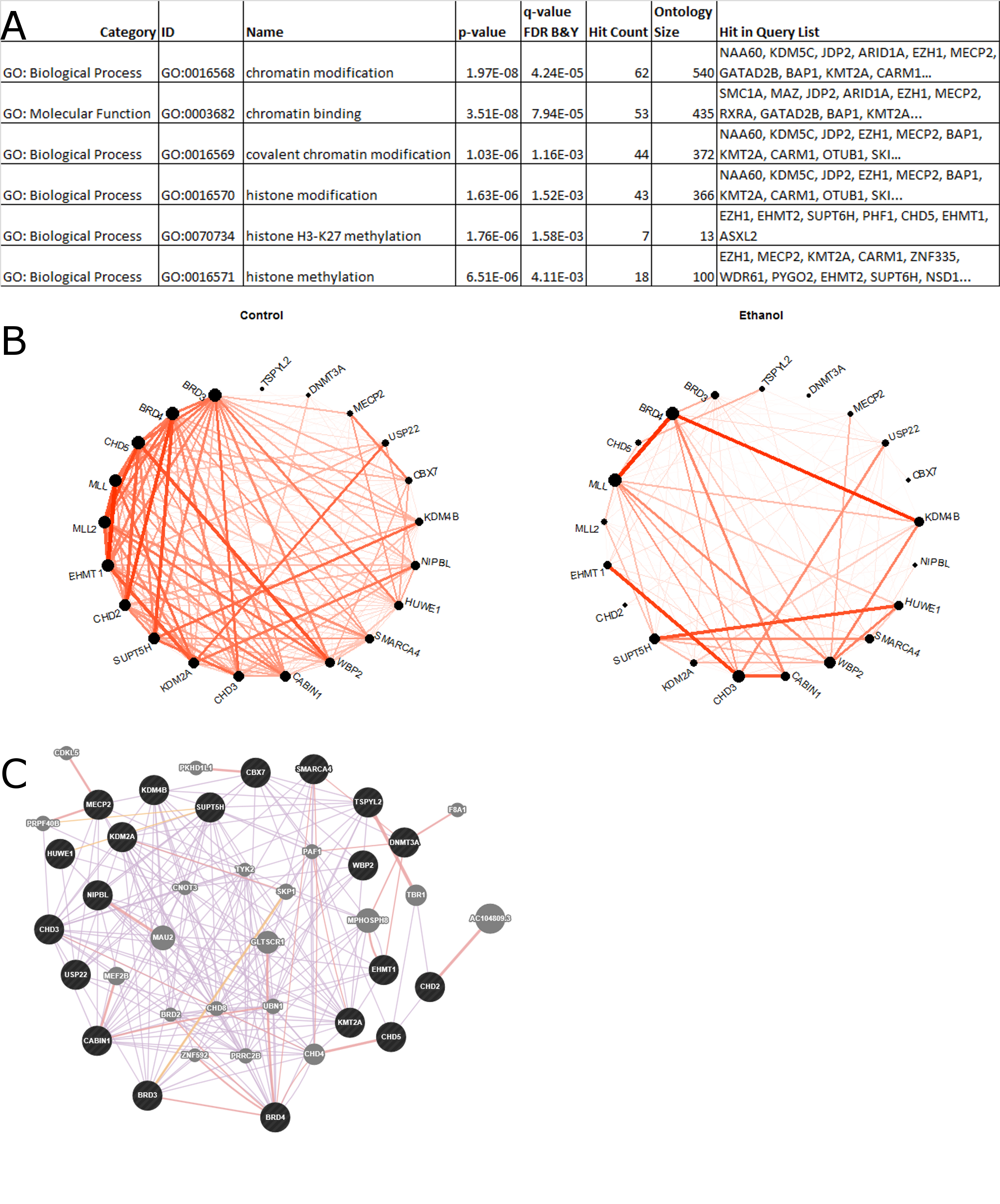

Supplement: Supplementary file 17 [file Data_Sheet_2.zip › Image 8.TIFF]

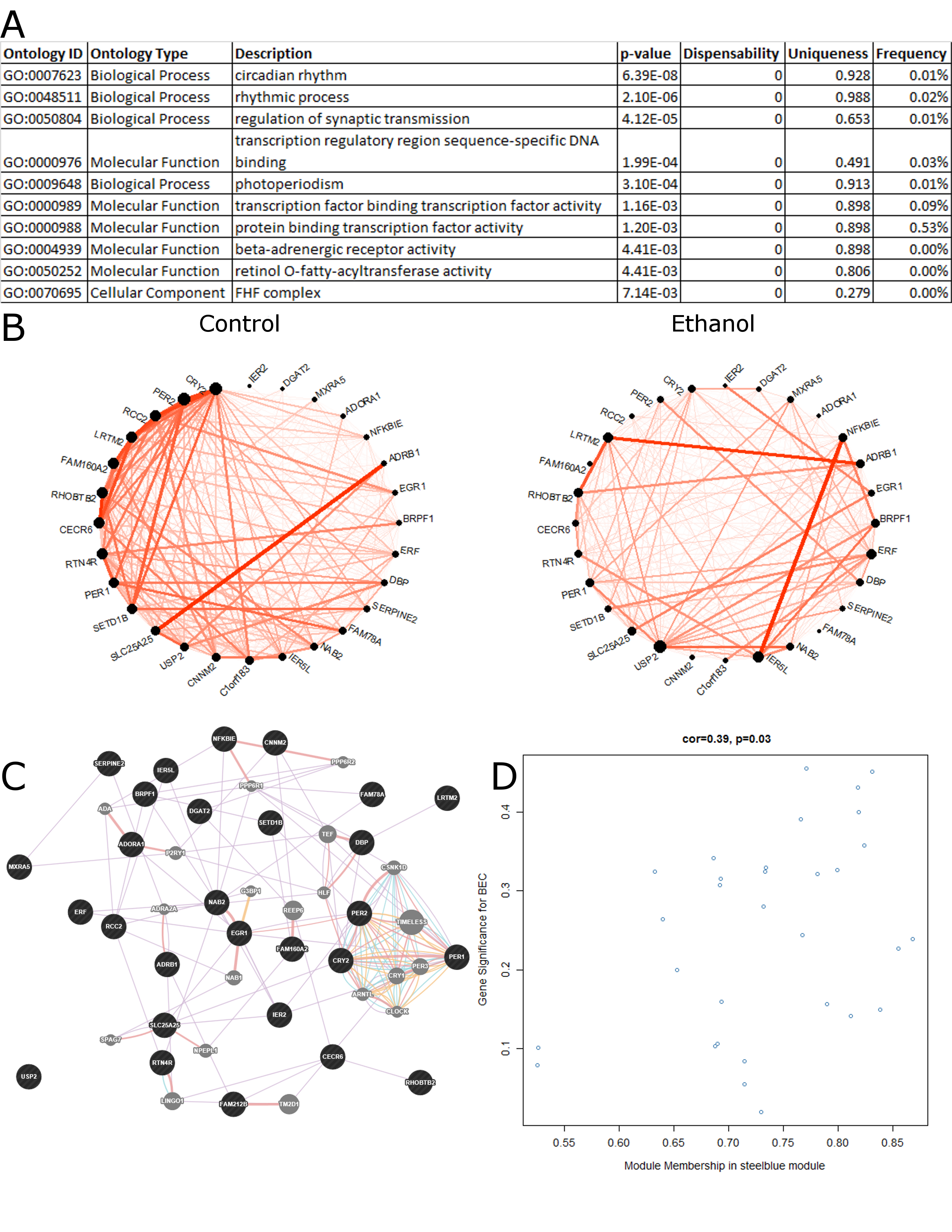

Supplement: Supplementary file 17 [file Data_Sheet_2.zip › Image 9.TIFF]
